# Cell Cycle and Cell Size Dependent Gene Expression Reveals Distinct Subpopulations at Single-Cell Level

**DOI:** 10.3389/fgene.2017.00001

**Published:** 2017-01-25

**Authors:** Soheila Dolatabadi, Julián Candia, Nina Akrap, Christoffer Vannas, Tajana Tesan Tomic, Wolfgang Losert, Göran Landberg, Pierre Åman, Anders Ståhlberg

**Affiliations:** ^1^Department of Pathology and Genetics, Sahlgrenska Cancer Center, Institute of Biomedicine, University of GothenburgGothenburg, Sweden; ^2^Center for Human Immunology, Autoimmunity and Inflammation, National Institutes of HealthBethesda, MD, USA; ^3^Department of Physics, University of MarylandCollege Park, MD, USA

**Keywords:** cell cycle, cell size, single-cell gene expression, machine learning, variable selection, random forests, cell subpopulations, cell transitions

## Abstract

Cell proliferation includes a series of events that is tightly regulated by several checkpoints and layers of control mechanisms. Most studies have been performed on large cell populations, but detailed understanding of cell dynamics and heterogeneity requires single-cell analysis. Here, we used quantitative real-time PCR, profiling the expression of 93 genes in single-cells from three different cell lines. Individual unsynchronized cells from three different cell lines were collected in different cell cycle phases (G0/G1 – S – G2/M) with variable cell sizes. We found that the total transcript level per cell and the expression of most individual genes correlated with progression through the cell cycle, but not with cell size. By applying the random forests algorithm, a supervised machine learning approach, we show how a multi-gene signature that classifies individual cells into their correct cell cycle phase and cell size can be generated. To identify the most predictive genes we used a variable selection strategy. Detailed analysis of cell cycle predictive genes allowed us to define subpopulations with distinct gene expression profiles and to calculate a cell cycle index that illustrates the transition of cells between cell cycle phases. In conclusion, we provide useful experimental approaches and bioinformatics to identify informative and predictive genes at the single-cell level, which opens up new means to describe and understand cell proliferation and subpopulation dynamics.

## Introduction

Cell proliferation is a tightly organized process that involves cell division and cell growth, where cell division can be divided into distinct cell cycle phases: G0, G1, S, G2, and M. Transitions through the phases are regulated by several layers of checkpoints and control mechanisms (Baserga, 1981; Lubischer, 2007; Bertoli et al., 2013; Grant et al., 2013). The molecular processes behind cell cycle progression have been dissected by numerous morphological studies on live or fixed single cells using a plethora of techniques to visualize components and processes during cell division. Many more investigations have been made on cells, sorted according to size, or artificially arrested at various cell cycle checkpoints. However, most of our knowledge about cell proliferation comes from studies that average data from large and mixed cell populations. Such data are only indirectly related to quantitative changes in cells at different states of division and growth. Analysis at the single-cell level can overcome most of these limitations. Detailed single-cell analyses have shown that transcript numbers fluctuate in individual cells, even in seemingly homogeneous populations (Raj et al., 2006), and that features of the typical or average cell in a population cannot be deduced from measurements on cell population samples (Bengtsson et al., 2005). Variations in transcript numbers allow cells to produce unique responses to internal and external cues that lead to defined paths of cell proliferation and differentiation (Levine et al., 2013). Recent development of single-cell analytical platforms opens up new possibilities to define the molecular profiles of cells at different states and to determine the importance of cell heterogeneity on cellular processes and cell fate decisions (Kalisky et al., 2011; Ståhlberg et al., 2011b; Sanchez and Golding, 2013; Shapiro et al., 2013).

Here, we employed single-cell gene expression profiling to describe the dynamic transition between cell proliferative states in three different cell lines using a panel consisting of 93 marker genes. Function of selected genes related to cell proliferation, cell cycle regulation, TP53 function, stemness, differentiation, cell signaling, and housekeeping functions (for gene details, see Table S1). We assessed cell division by collecting cells in the G0/G1, S and G2/M phases, and cell growth by selecting small and large cells in respective cell cycle phase. In contrast to cell population data, single-cell data are reported as transcripts per cell without any further normalization (Ståhlberg et al., 2013), allowing total transcript levels to be determined and compared between cell states (Sanchez and Golding, 2013). To determine if, and to what degree, the gene expression profile of individual cells were associated with cell division and growth we applied the random forests algorithm (Hastie et al., 2009; Gareth et al., 2013), which is a supervised machine learning approach. By applying variable selection, a recursive feature elimination (RFE) scheme (James et al., 2013; Candia et al., 2015), we were able to identify the genes with strongest cell proliferation association and to define distinct subpopulations. Finally, we calculated a cell cycle index based on the most predictive genes that allowed us to visualize and biologically interpret cell cycle progression.

## Materials and methods

### Cell culture

All cell lines were cultured at 37°C and in 5% CO_2_. The myxoid liposarcoma cell line MLS 402-91 was cultured in RPMI 1640 GlutaMAX medium supplemented with 10% fetal bovine serum, 100 U/mL penicillin, and 100 μg/mL streptomycin (all Life Technologies). Cells were passaged with 0.25% trypsin and 0.5 mM EDTA (both Life Technologies). The breast cancer cell line MCF7 was cultured in DMEM medium supplemented with 2 mM L-glutamine, 1% penicillin/streptomycin (all PAA Laboratories), 10% fetal bovine serum (Lonza), and 1% non-essential amino acids (Sigma-Aldrich). MCF7 cells were passaged with 0.05% trypsin-EDTA (PAA Laboratories). Mesenchymal stem cells (MSC) derived from human embryonic stem cells (hES-MP 002.5, Takara Bio), were cultured in DMEM GlutaMAX, supplemented with 10% fetal bovine serum, 100 U/mL penicillin, 100 μg/mL streptomycin, and 4 ng/mL fibroblast growth factor 2 (all Life Technologies) as described (Karlsson et al., 2009). MSCs were passaged with TrypLE Select (Life Technologies). Dissociation enzyme inactivation was performed using complete medium, containing fetal bovine serum for all cell lines. Cell cultures were confirmed as mycoplasma-free using the Mycoplasma PCR Detection Kit (Applied Biological Materials).

### Fluorescent activated cell sorting

Vybrant DyeCycle violet stain (Life Technologies) and CellVue Claret far red dye (Sigma-Aldrich) were used to stain genomic DNA and membrane lipids, respectively. Suspension of 10^6^ cells in 1 mL Hanks' balanced salt solution (Life Technologies) was first stained with Vybrant DyeCycle violet stain (5 μM, final concentration) at 37°C for 30 min. Then, 1 mL CellVue Claret far red dye diluted in diluent C (Sigma-Aldrich, 3.3 μM, final concentration) was added followed by an incubation step at 37°C for 5 min. Staining was inactivated by complete medium and the cells were finally resuspended in Hanks' balanced salt solution.

G1/S cell cycle arrest was performed using a double thymidine block (Sigma-Aldrich). Thymidine (2 mM, final concentration) was added to 25–30% confluent cells for 18 h. Cells were then released by addition of fresh medium without thymidine. Finally, after 9 h cells were re-exposed to thymidine for additional 17 h. Complete cell cycle arrest was confirmed by Vybrant DyeCycle violet staining followed by fluorescence activated cell sorting analysis.

Cell aggregates were removed by filtering with a 40 μm cell strainer (BD Biosciences) and single cells were sorted with a BD FACSAria II (BD Biosciences) into 96-well-plates (Life Technologies), each well-containing 5 μL 1 mg/mL bovine serum albumin (Thermo Scientific; Svec et al., 2013). Collected single cells were frozen on dry ice and kept at −80°C until subsequent analysis. Gating strategies for cell size and cell cycle phase are shown in Figure S1. The cell size/cell volume was estimated from the average CellVue Claret far red signal, assuming a spherical cell shape. All single-cells from respective biological condition were collected from an individual culture, to minimize batch-to-batch differences as described (Wills et al., 2013).

### Single-cell gene expression profiling

Reverse transcription was performed with SuperScript III (Life Technologies). Lysed single cells, 0.5 mM dNTPs (Sigma-Aldrich), 5.0 μM Oligo(dT_12−18_), and 5.0 μM random hexamers (both Life Technologies) were incubated in 6.5 μL at 65°C for 5 min. Next, 50 mM Tris–HCl, 75 mM KCl, 3 mM MgCl_2_, 5 mM dithiothreitol, 10 U RNaseOut, and 50 U SuperScript III (all Life Technologies) were added to a final volume of 10 μL. Final reaction concentrations are shown. Reverse transcription was performed at 25°C for 5 min, 50°C for 60 min, 55°C for 10 min, and terminated by heating to 70°C for 15 min. All samples were diluted to 30 μL with water.

Targeted cDNA preamplification was performed with the iQ Supermix (BioRad) in 50 μL reactions. Each reaction contained 10 or 15 μL diluted cDNA and 40 nM of each primer. Primer sequences are shown in Table S1. Optimization and validation of good performing qPCR assays and preamplification are described elsewhere (Ståhlberg and Bengtsson, 2010; Andersson et al., 2015). The temperature profile was 95°C for 3 min followed by 20 cycles of amplification (95°C for 20 s, 60°C for 3 min, and 72°C for 20 s). All preamplified samples were chilled on ice and diluted 1:20 in TE-buffer (pH 8.0; Life Technologies). Preamplification was performed as two separate reactions for each single cell, each containing half of the assays. The products of the two reactions were pooled after preamplifciation. Reproducibility and efficiency of the preamplification were evaluated by standard curve analysis using cDNA from MLS 402-91 (Figure S2). The overall preamplification efficiency was assessed using five different cDNA concentrations (*n* = 4) generated from 0.04, 0.2, 1, 5, 25 ng total RNA, respectively. The average cycle of quantification value of all genes expressed in four or more dilutions were used to determine the overall preamplification efficiency.

The BioMark real-time PCR system with 96 × 96 dynamic arrays (Fluidigm) was used for gene expression profiling according to the manufacturer's instructions. The 5 μL sample reaction mixture contained 1X SsoFast EvaGreen Supermix (BioRad), 1X ROX (Life Technologies), 1X GE Sample Loading Reagent (Fluidigm), and 2 μL diluted preamplified cDNA. The 5 μL primer reaction contained 1X Assay Loading Reagent (Fluidigm) and 5 μM of each primer. Preamplification and qPCR were performed with the same primers (Table S1). The chip was first primed with the NanoFlex IFC Controller (Fluidigm) and then loaded with the sample and primer reaction mixtures. The cycling program was 3 min at 95°C for polymerase activation, followed by 40 cycles of amplification (96°C for 5 s and 60°C for 20 s). After qPCR, all samples were analyzed by melting curve analysis (60–95°C with 0.33°C per s increment). All assays were confirmed to generate correct PCR product length by agarose gel electrophoresis. Data pre-processing was performed with GenEx (v.6, MultiD) as described (Ståhlberg et al., 2013). Briefly, samples with aberrant melting curves were removed and cycle of quantification values larger than 25 were replaced with 25. Data were transformed to relative quantities assuming that a cycle of quantification value of 25 equals one molecule. Missing data were replaced with 0.5 molecules. All data were calculated per cell if not stated otherwise. For all data analysis we assumed 100% PCR efficiency. The impact of the chosen cut-off value and applied PCR efficiency had negligible effect on downstream analysis.

### Immunofluorescence

MLS 402-91 and MCF-7 cells were seeded on Millicell EZ SLIDE 4-well-glasses (Merck Millipore). After 24 h, cells were rinsed with phosphate buffer saline (Life Technologies) and fixed in 3.7% formaldehyde for 5 min (Sigma-Aldrich), washed three times with phosphate buffer saline and permeabilized in AB buffer (phosphate buffer saline supplied with 1% bovine serum albumin and 0.5% Triton X, Sigma-Aldrich). Cells were stained with anti-MCM6 antibody (HPA004818 rabbit, diluted 1:50, Sigma-Aldrich). Detection was performed with a Cy3 conjugated secondary antibody (PA43004, diluted 1:1000, GE Healthcare Life Sciences). Slides were mounted using Prolong Gold anti-fade with 4′,6-diamidino-2-phenylindole (Life Technologies). Cellular fluorescence was imaged using a Zeiss Axioplan 2 microscope (Zeiss). Relative protein level per cell was estimated using Volocity 3D Image Analysis Software (PerkinElmer).

### Single-cell data analysis and statistics

Principal component analysis, hierarchical clustering, and Kohonen self-organizing maps were performed in GenEx software using autoscaled gene expression data as described (Ståhlberg et al., 2011a). The Ward's algorithm and Euclidean distance measure were applied for hierarchical clustering. Parameters for Kohonen self-organizing maps were: 3–4 × 1 map, 2 neighbors, 0.4 learning rate, and 150 iterations. The resulting clusters were not sensitive to parameter choice.

A random forests algorithm was implemented to pairwise classify different cell cycle phases and cell sizes. Two cell states were compared at a time. Random forests are collections of decision trees. At the top-most level of each decision tree, all genes are scanned one by one, to determine the best gene, and corresponding gene expression threshold to optimally partition the original cells into two branches. The optimal partition is algorithmically determined based on the minimization of a quality function such as the cross-entropy or the Gini index (Hastie et al., 2009; Gareth et al., 2013), which aim to increase the class purity of each branch. Subsequently, each branch is considered for further separation based on the expression values of other genes. The process continues until the full decision tree is grown in such a manner that each of its leaves, i.e., the endpoint of each branch, contains cells of a single class. To generate robust solutions and avoid data overfitting, additional parameters are usually incorporated to the model in order to either limit the length of the tree (or, alternatively, the size of the nodes that can undergo further branching) or to prune the tree. In this context, a popular technique is to generate a so-called random forest that contains a large number of partially decorrelated trees built out of bootstrapped samples from the original data set. Compared to single decision trees, random forests are less intuitive, since they lack a direct visualization of the structure and relations among predictor genes, but random forests are more powerful and robust. In this study, we implemented a random forest analysis using the random Forest (v4.6-10) package in R. This implementation uses the decrease of Gini index impurity as a splitting criterion and selects the splitting predictor from a subset of predictors, randomly chosen at each split. Each random forest consisted of 1000 trees. For each random forest we scanned the size of the predictor subset in the full range from one to the total number of predictors and selected the smallest subset that minimized the out-of-bag error. The so-called out-of-bag error is calculated from predictions on out-of-bag instances, i.e., those cells that have not been used in building a particular tree. Moreover, in order to assess model variance, for each class comparison we generated ensembles consisting of 100 different random forests. Only genes with detectable expression in at least 50% of the cells in at least one cell class were included in our analysis. We report averages and standard deviations calculated over these random forest ensembles throughout.

Cell classification performance can be quantified by several measures. In addition to the out-of-bag error, another measure is the balanced accuracy. The balanced accuracy is the classification accuracy averaged over all classes, where the classification accuracy for each class is the percentage of cells in the class that are correctly classified by the random forest. Yet another measure is Fisher's *p*-value obtained by applying Fisher's exact test on the confusion matrix, which consists of the number of correctly and incorrectly classified cells in each class. Moreover, we also computed the so-called gene importance, a quantitative measure of the impact of the gene on the node purity.

To address the question of which, and how many, genes are needed to best separate two classes we applied a recursive feature elimination (RFE) scheme, a standard approach for feature selection (Tarca et al., 2007; Candia et al., 2013). In the first RFE cycle, we generated a random forest ensemble using all (N) genes and computed classification statistics, including confusion matrices with associated Fisher's *p*-value, balanced accuracy, out-of-bag error, and gene importance. We determined the least significant gene based on gene importance and removed it. Then, in the second RFE cycle we used the remaining *N*–1 genes and repeated the random forest analysis to eliminate the second least significant gene. The procedure was subsequently iterated until one gene was left. By comparing the classification performance across all RFE cycles we could then determine the number of genes in the optimal gene signature. We verified that, for this optimal gene signature, the out-of-bag error and Fisher's *p*-value were minimized, while the balanced accuracy was maximized. The intended redundancy of separately considering three classification performance metrics allowed us to ensure the robustness of the optimally obtained gene signature.

The most predictive genes identified by RFE was used to calculate a cell cycle index as the sum of all G1 to S and/or G2/M upregulated genes subtracted by the sum of all G1 to S and/or G2/M downregulated genes divided by the number of genes used. The lg2 expression value of each gene was used.

## Results

Gene expression and cell heterogeneity of proliferating cells were studied by fluorescence activated cell sorting combined with single-cell gene expression profiling. Three different cell lines were investigated: a genetically stable myxoid liposarcoma cell line (MLS 402-91) (Aman et al., 1992); a breast cancer adenocarcinoma derived cell line (MCF7; Soule et al., 1973) and mesenchymal stem cells (MSC) differentiated from an embryonic stem cell line (Karlsson et al., 2009). Cells were stained with lipid and DNA binding dyes, visualizing cell size, and DNA content. Utilizing this double-labeling approach we collected small and large cells in the G0/G1, S, and G2/M phases (Figure S1). DNA staining cannot distinguish between G0 and G1 phase cells, or between G2 and M phase cells. We refer the G0/G1 phase as G1 phase only, since few G0 cells are expected in our continuously passaged cell cultures. The average volume ratio between large and small collected cells was 2.8 for MLS 402-91, 2.5 for MCF7, and 4.5 for MSC (Figure S1). Expression of 93 genes were analyzed in each cell using reverse transcription quantitative real-time PCR. One gene (*FUS*) was assessed by two assays. Assay information and gene function are shown in Table S1. All basic data, including number of positive cells expressing each gene and mean single-cell expression with standard deviation, are shown in Table S2. We tested the reproducibility of our data by collecting individual MLS 402-91 cells in the G1, S, and G2/M phases without any cell size selection in an independent experiment.

### Total transcript level correlates with cell cycle phase at the single-cell level

**Figure 1:**
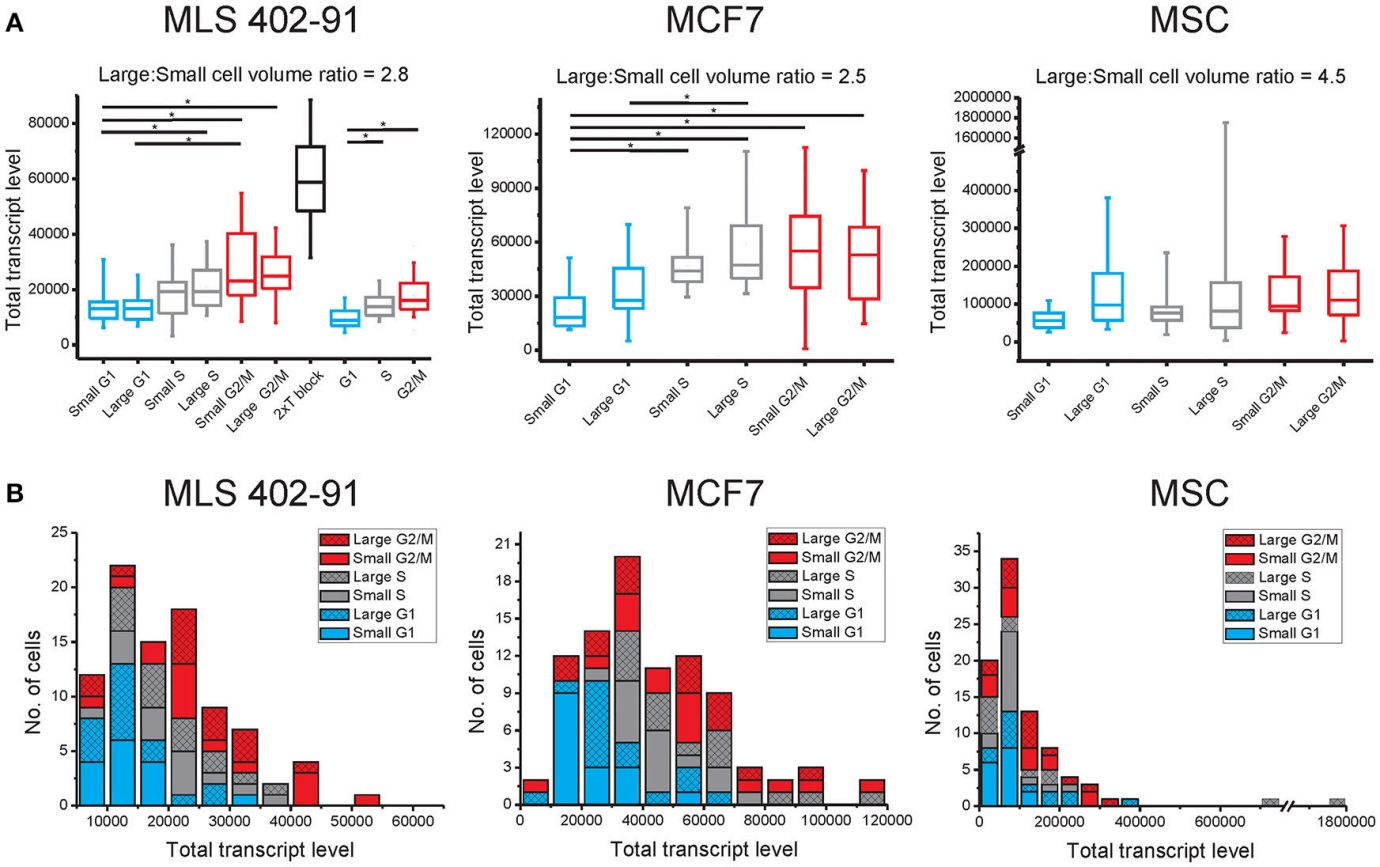
**Total transcript levels are mainly cell cycle phase dependent**. **(A)** The total transcript level for small and large cells in the G1 (blue), S (gray), and G2/M (red) phases are shown (MLS 402-91: *n*_small−G1_ = 15, *n*_large−G1_ = 16, *n*_small−S_ = 15, *n*_large−S_ = 15, *n*_small−G2/M_ = 15, *n*_large−G2/M_ = 15; MCF7: *n*_small−G1_ = 16, *n*_large−G1_ = 15, *n*_small−S_ = 15, *n*_large−S_ = 15, *n*_small−G2/M_ = 15, *n*_large−G2/M_ = 16, and MSC: *n*_small−G1_ = 16, *n*_large−G1_ = 13, *n*_small−S_ = 16, *n*_large−S_ = 12, *n*_small−G2/M_ = 14, *n*_large−G2/M_ = 15). In addition, G1/S phase arrested MLS 402-91 cells with any cell size were analyzed, using a double thymidine block (*n* = 61). As a separate experiment, MLS 402-91 cells were collected and analyzed based on cell cycle phase only (*n*_G1_ = 30, *n*_S_ = 29 and *n*_G2/M_ = 30). Box-Whisker plots are shown; the box ranges between the 25 and 75% and the whiskers range between the 5 and 95% of all data. ^*^indicate 95% significance using the Mann-Whitney *U*-test with Holm-Bonferroni correction for multiple testing. **(B)** Distribution of total transcript levels among individual cells in MLS 402-91, MCF7, and MSC. The total transcript level per cell is calculated as the sum of all measured transcript for all 93 genes.

Transcript numbers were measured per single cell without any further normalization between cells (Ståhlberg et al., 2011a, 2013). Hence, the total transcript level could be calculated as the sum of all measured transcripts per cell. Figure 1A and Table 1 show that the total transcript level correlated with cell cycle phase, but not with cell size. In MLS 402-91 the total transcript level reached maximum in G2/M phase cells with about two-fold higher levels compared to G1 phase cells. In MCF7 the total transcript level reached maximum in S phase cells and remained at the same level in G2/M phase cells. MSC only displayed a weak correlation between total transcript level and cell cycle phase.

**Table 1:** **Spearman's correlation coefficient between total transcript level and cell proliferation parameters at single-cell level**.

	**MLS 402-91**	**MCF7**	**MSC**
Cell cycle phase combined with cell size	0.27^*^	0.34^**^	0.28^**^
Cell cycle phase	0.51^**^	0.47^**^	0.23^*^
Cell size	0.03		0.19

* p < 0.05,

** *p < 0.01*.

The total transcript level varied highly between individual cells (Figure 1B). The distributions were skewed with few cells containing high total transcript levels. The total transcript level was 17, 120, and 820 times higher in the cell with highest total transcript level compared to the cell with lowest total transcript level in MLS 402-91, MCF7, and MSC, respectively (all cells included). Correlation analysis between transcript levels of individual genes at single-cell level showed positive correlations between most genes: 74% in MLS 402-91 (total number of comparisons = 4278), 85% (total number of comparisons = 3081) in MCF7 and 90% (total number of comparisons = 3486) in MSC. Consequently, cells with high total transcript level also displayed elevated transcript numbers of most individual genes.

### Identification of genes with cell cycle phase and cell size dependent expression

**Figure 2:**
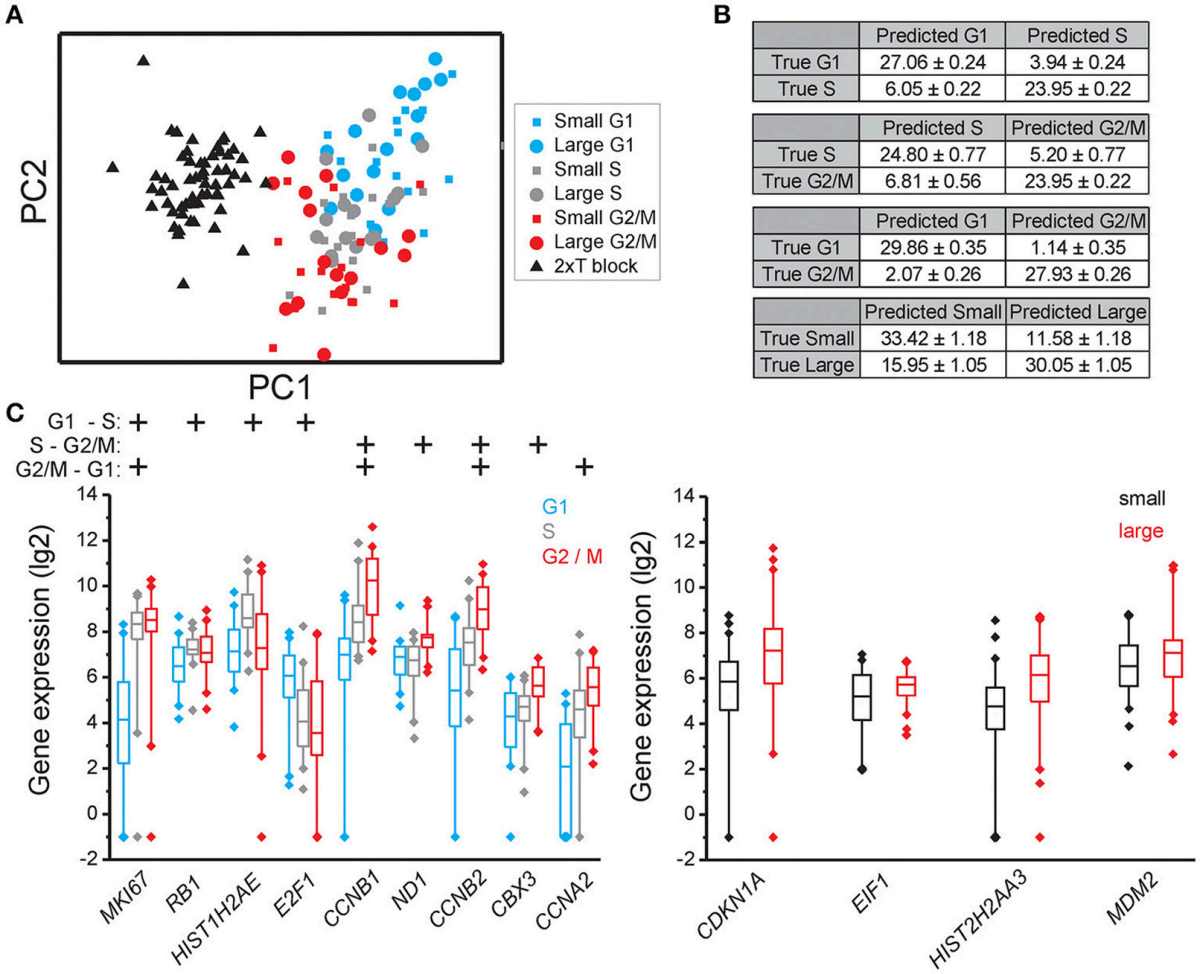
**Cell cycle phase and cell size dependent gene expression in MLS 402-91**. **(A)** PCA of small and large MLS 402-91 cells in the G1, S, and G2/M phases. Note that the double thymidine treated cells (T block) show a completely different expression profile than non-treated cells. Each dot, square, and triangle represents a single cell. **(B)** Confusion matrices of cell classifications using the random forests algorithm. Fisher's exact test was used to calculate significance (*p* < 0.0001) for all matrices. **(C)** Box-Whisker plots for the genes with highest importance to classify cell cycle phase and cell size using the random forests algorithm. The box ranges between the 25 and 75%, the whiskers range between the 5 and 95% of all data and outliers are indicated as diamonds.

**Figure 3:**
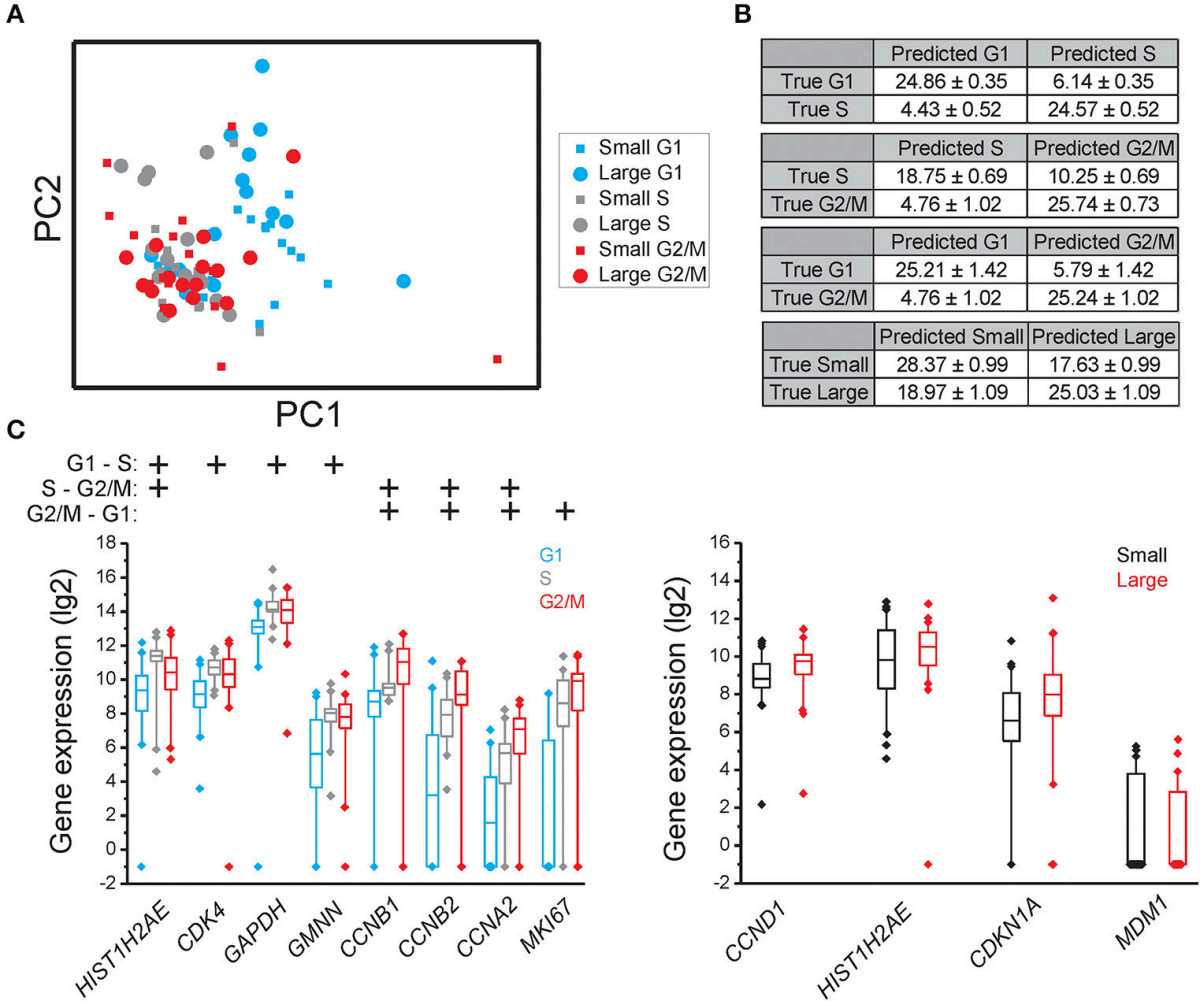
**Cell cycle phase and cell size dependent gene expression in MCF7**. **(A)** PCA of small and large MCF7 cells in the G1, S, and G2/M phases. Each dot and square represents a single cell. **(B)** Confusion matrices of cell classifications using the random forests algorithm. Fisher's exact test was used to calculate significance (*p* < 0.0001) for all matrices. The confusion matrix of small compared to large cells was not significant. **(C)** Box-Whisker plots for the genes with highest importance to classify cell cycle phase and cell size using the random forests algorithm. The box ranges between the 25 and 75%, the whiskers range between the 5 and 95% of all data and outliers are indicated as diamonds.

**Figure 4:**
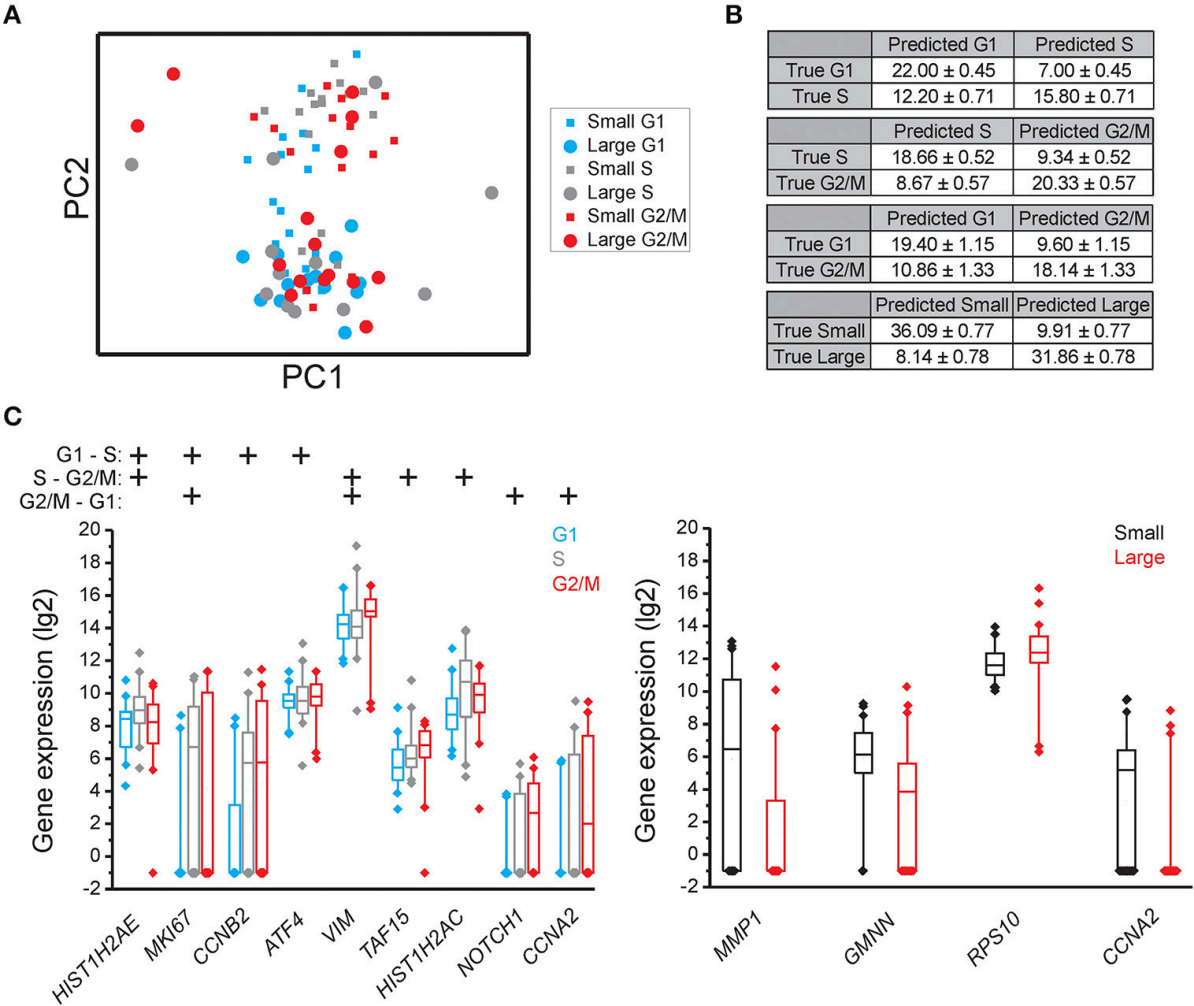
**Cell cycle phase and cell size dependent gene expression in MSC**. **(A)** PCA of small and large MSC cells in the G1, S, and G2 phases. Each dot and square represents a single cell. **(B)** Confusion matrices of cell classifications using the random forests algorithm. Fisher's exact test was used to calculate significance (*p* < 0.05) for all matrices. **(C)** Box-Whisker plots for the genes with highest importance to classify cell cycle phase and cell size using the random forests algorithm. The box ranges between the 25 and 75%, the whiskers range between the 5 and 95% of all data and outliers are indicated as diamonds.

Principal component analysis (PCA) showed that individual cells partly clustered based on their cell cycle phase in all three cell lines (MLS 402-91 in Figure 2A, MCF7 in Figure 3A, and MSC in Figure 4A), but only MSC displayed cell size depended clustering. However, large overlaps between cells of different cell cycle phases and cell sizes were observed for all cell lines. Double thymidine treated MLS 402-91 cells showed a completely divergent expression profile compared to non-treated G1, S, or G2/M phase cells, demonstrating that artificial cell synchronization result in severe and unintended side effects (Figure 2A).

To determine if individual cells can be correctly classified into cell cycle phase or cell size based on their gene expression profile we applied the random forests algorithm, a machine-learning approach based on decision trees. As a classifier, a decision tree is a hierarchically organized structure that optimally can separate cell cycle phases and cell sizes (see Section Materials and Methods for details). Figures 2B, 3B, 4B show how well-cell cycle phase and cell size could be distinguished using a multi-gene signature at the single-cell level. In MLS 402-91, we obtained best classification comparing G2/M with G1 phase cells, while the classifications between other cell cycle phases were less efficient (Figure 2B). For example, 29.86 ± 0.35 out of 31 MLS 402-91 cells were correctly classified as G1 phase cells, while 1.14 ± 0.35 G1 phase cells were falsely predicted to be G2/M phase cells. The ability to classify MCF7 cells was similar (Figure 3B). The gene expression profile was less predictive to classify cell size than cell cycle phase in both MLS 402-91 and MCF7 cells (Figures 2B, 3B). Similar gene expression profiles and classifications were also observed for the independent MLS 402-91 data set (Figure S3). The gene expression profile of individual MSC was less predictive for cell cycle phases compared to the two other cell lines, but the ability to classify cell size was more efficient in MSC (Figure 4B). We also compared small and large cells within respective cell cycle phase, but no distinct cell size dependency was found in any of the three cell lines (data not shown). The random forests approach also allowed us to rank the individual genes based on their importance in the classification (Figure S4). Figures 2C, 3C, 4C show the genes with strongest cell cycle phase and cell size dependent expression. Even if the median expression level of these predictive genes correlated well with their ability to classify cell cycle phase or cell size, individual cells showed highly variable, and overlapping gene expression (Figures 2C, 3C, 4C).

### Identification of predictive genes and cell line specific subpopulations

**Figure 5:**
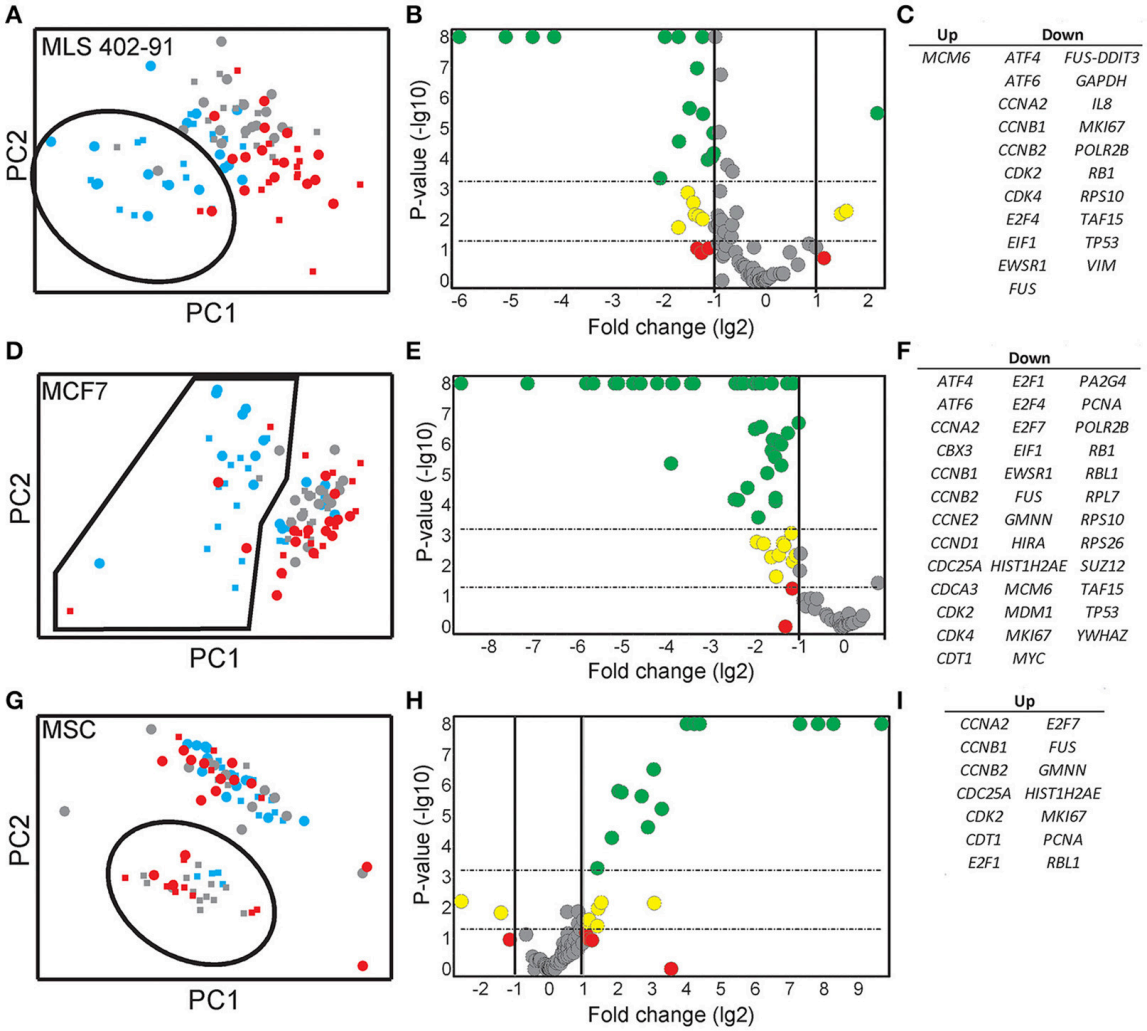
**Identification and characterization of distinct subpopulations**. **(A)** A MLS 402-91 subpopulation (encircled) was defined using PCA and RFE identified genes (*MKI67, RB1, HIST1H2AE, CCNB1, CBX3, ND1, GAPDH, CCNB2*, and *E2F1)*. Individual small (squares) and large (dots) MLS 402-91 cells in G1 (blue), S (gray), and G2/M (red) phase are shown. **(B)** The volcano plot shows regulation and significance of all analyzed genes, comparing the defined G1 subpopulation and the remaining G1 phase cells. Dunn-Bonferroni correction for multiple testing (*p* < 0.00054) was applied using 95% significance. Red (*p* > 0.05), yellow (0.05 > *p* > 0.00054), and green (*p* < 0.00054) dots indicate at least two-fold regulated genes. **(C)** All significantly MLS 402-91 regulated genes identified in the volcano plot are listed. **(D)** A MCF7 subpopulation (encircled) was defined using PCA and RFE identified genes (*HIST1H2AE, CCNB1, CDK4, GMNN, CCNB2*, MKI67, RPS10, *RPL7*, and *EIF1*). Individual small (squares) and large (dot) MCF7 cells in G1 (blue), S (gray), and G2/M (red) phase are shown. **(E)** The volcano plot shows regulation and significance for all analyzed genes in MCF7, comparing the defined G1 subpopulation and the remaining G1 phase cells. Dunn-Bonferroni correction for multiple testing (*p* < 0.00062) was applied using 95% significance. Red (*p* > 0.05), yellow (0.05 > *p* > 0.00062) and green (*p* < 0.00062) dots indicate at least two-fold regulated genes. **(F)** All significantly MCF7 regulated genes identified in the volcano plot are listed. **(G)** A MSC subpopulation (encircled) was defined using PCA and RFE identified genes (*HIST1H2AE, MKI67, ATF4, YWHAZ*, E2F4, TAF15, *RB1, CCNA2, NOTCH1, CCNB1*, and *VIM*). Individual small (squares) and large (dot) MCF7 cells in G1 (blue), S (gray), and G2/M (red) phase are shown. **(H)** The volcano plot shows regulation and significance for all analyzed genes in MSC, comparing the defined subpopulation and the remaining cells. Dunn-Bonferroni correction for multiple testing (*p* < 0.0006) was applied using 95% significance. Red (*p* > 0.05), yellow (0.05 > *p* > 0.0006), and green (*p* < 0.0006) dots indicate at least two-fold regulated genes. **(I)** All significantly MSC regulated genes identified in the volcano plot are listed.

Expression data for all genes were used in the random forests classification algorithm to predict cell cycle phase and cell size. To determine if a similar prediction model could be generated with fewer genes, we applied a recursive feature elimination (RFE) approach. In RFE, the least informative gene is eliminated from the random forests analysis. This procedure is repeated until only one gene remains. Figure S5 shows how well the random forests algorithm performed with decreasing number of genes. We found that expression data from the following gene sets were almost as accurate as the complete gene panel in classifying cell cycle phase in MLS 402-91: G1 vs. S: *MKI67, RB1, E2F1, HIST1H2AE*, and *CCNB1*; S vs. G2/M: *CCNB1, CBX3*, and *ND1* and G2/M vs. G1: *MKI67, GAPDH, CCNB1*, and *CCNB2*. The gene lists are ordered with the most predictive gene listed first. Refined PCA using only these nine predictive genes revealed a distinct subpopulation that was not clearly visible using all genes (Figure 5A). The same subpopulation was also identified using other algorithms, including hierarchical clustering and Kohonen self-organizing maps (Figure S6). This new subpopulation mainly consisted of G1 cell cycle phase cells and was characterized by upregulation of *MCM6* and downregulation of 21 other genes, mainly cell cycle related genes (Figures 5B,C). We refer to this subpopulation as the G1′ subpopulation. The total transcript level in the G1′ subpopulation was on average 32% lower compared to the other G1 phase cells (*p* < 0.01, Mann-Whitney *U*-test), suggesting a distinct G1 cell state with low transcriptional activity. We also confirmed the presence of the same G1 subpopulation with almost an identical gene expression profile in the independent MLS 402-91 data set (Figure S7).

In MCF7, the following sets of predictive genes were identified by RFE: G1 vs. S phase: *HIST1H2AE, CCNB1, CDK4*, and *GMNN*; S vs. G2/M phase: *CCNB1, CCNB2*, and *HIST1H2AE* and G2/M vs. G1 phase: *MKI67, CCNB1, RPS10, RPL7*, and *EIF1*. Refined PCA revealed a G1 subpopulation with similar characteristics as the G1′ subpopulation found in MLS 402-91 (Figures 5D–F). The existence of the MCF7 defined G1′ subpopulation was confirmed by hierarchical clustering and Kohonen self-organizing maps (data not shown). The total transcript level was 47% lower in the G1′ subpopulation compared to the other G1 phase cells (*p* < 0.01, Mann-Whitney *U*-test). One gene, *MCM6*, displayed opposite regulation in the G1′ subpopulation in MCF7 compared to MLS 402-91. The variable and divergent *MCM6* expression prompted us to analyze its protein expression. Immunofluorescence analysis showed variable MCM6 protein expression in both MLS 402-91 and MCF7 with somewhat higher variability in MCF7 cells (Figure S8).

In MSC, RFE generated the following sets of predictive genes: G1 vs. S phase: *HIST1H2AE, MKI67, ATF4*, and *YWHAZ*; S vs. G2/M phase: *HIST1H2AE, E2F4, TAF15*, and *RB1* and G2/M vs. G1 phase: *CCNA2, NOTCH1, CCNB1*, and *VIM*. In contrast to MLS 402-91 and MCF7, MSC displayed a distinct subpopulation of small S and G2/M phase cells that was characterized by upregulated cell proliferation genes (Figures 5G–I). The existence of this MSC specific subpopulation was also confirmed by other algorithms (data not shown).

### Cell cycle progression can be visualized by a cell cycle index based on gene expression

**Figure 6:**
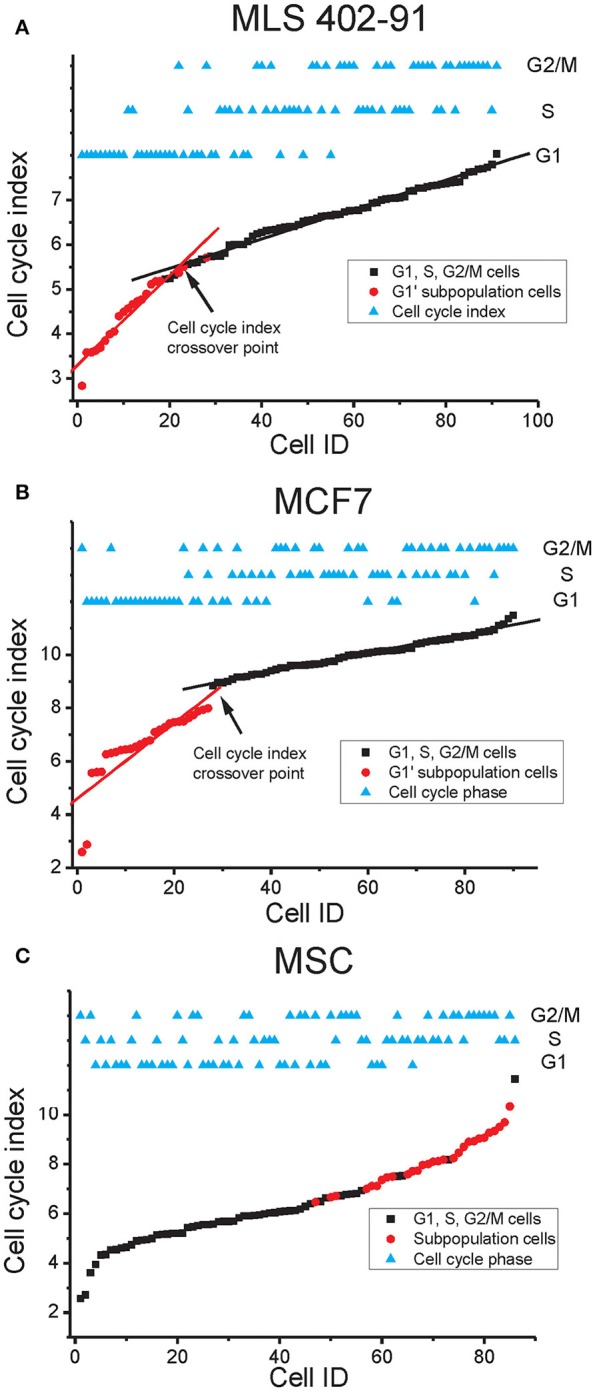
**Cell cycle index**. The cell cycle index of each cell is shown in relation to its cell cycle phase. Subpopulation cells identified in Figure 5 are also indicated. **(A)** The MLS 402-91 index was calculated as: (*MKI67* + *RB1* + *HIST1H2AE* + *CCNB1* + *CBX3* + *ND1* + *GAPDH* + *CCNB2* – *E2F1*)/9. The lg2 expression value of each gene was used. The cell cycle index crossover point where the index enters a plateau is indicated. The linear fits are shown to guide the eye. **(B)** The MCF7 index was calculated as: (*HIST1H2AE* + *CCNB1* + *CDK4* + *GMNN* + *CCNB2* + *MKI67* + *RPS10* + *RPL7* +*EIF1*)/9. The lg2 expression value of each gene was used. The cell cycle index crossover point where the index enters a plateau is indicated. The linear fits are shown to guide the eye. **(C)** The MSC index was calculated as: (*HIST1H2AE* + *MKI67* + *ATF4* + *YWHAX* + *E2F4* + *TAF15* + *RB1* + *CCNA2* + *NOTCH1* + *CCNB2* + *VIM*)/11. The lg2 expression value of each gene was used.

Multi-gene profiles are usually hard to visualize and interpret. Hence, we calculated and plotted a cell cycle index based on the expression of all cell cycle regulated genes identified by RFE for each cell line (Figure 6). The index correlated with the cell cycle progression for all three cell lines, where G1 phase cells showed low indexes, while G2/M phase cells displayed high indexes. The cell cycle index varied most between individual G1 phase cells in MLS 402-91 and MCF7, where a distinct index crossover point could be identified for cells in the transition from G1 to S phase. In contrast, MSC showed a different pattern with a more uniform G1 to S phase transition. The cells in the G1′ subpopulations identified in MLS 402-91 and MCF7 displayed the lowest cell cycle indexes, while the cells in the subpopulation defined in MSC showed the highest indexes.

## Discussion

The mechanisms governing cell growth and division of mammalian cells have long been a subject of intense research. Many of the decisive regulatory events occur by post translational modifications of pre-existing proteins (Pagliuca et al., 2011), but underlying this regulatory level is also synchronized *de novo* production of cell cycle regulated components. A large number of genes have been reported to be timely transcribed as part of cell cycle progression (Sun et al., 2007; Simmons Kovacs et al., 2008; Muller and Engeland, 2010). Here, we have taken advantage of emerging technology to study gene expression profiles in single cells of different cell cycle phases and of different cell sizes. To date, most studies aimed at cell cycle regulated gene transcription were based on large cultures and artificial cell synchronization. We and others (Cooper, 2002, 2003) have observed that standard synchronization strategies affect cell states in unintended ways as they cause cell stress and abnormal expression profiles (Figures 1A, 2A). Our approach to collect unsynchronized individual cells avoids these issues and our data clearly demonstrate some of the benefits using single-cell analysis. Both the observed cell-to-cell variability and the identified subpopulations would have been challenging to study at cell population level.

Traditional expression analysis usually involves normalization processes before samples can be compared. Normalization assumes that selected house-keeping genes, i.e., reference genes, or the total amount of transcripts is essentially identical across samples. However, single-cell RT-qPCR data are reported as transcripts per cell without the need of additional normalization between cells, which enable us to calculate the total transcript level of all analyzed genes (Ståhlberg et al., 2011a, 2013). This strategy is possible, since single cells are analyzed directly without any extraction steps. Our data show that the assumption of equal total transcription levels between individual cells is not valid. Instead, we observed that the total transcript level correlated with the cell cycle phase (Table 1). This was further tested by analyzing an additional published single-cell astrocyte data set generated directly from dissociated mice brains (Figure S9; Rusnakova et al., 2013). Taken together, our data show a considerable cell-to-cell variation in total transcript levels where most genes are positively correlated. In addition, only a minority of cells displayed elevated total transcript levels. Consequently, these few cells expressed high number of transcripts of most genes. The absolute values of the calculated total transcript levels are dependent on the applied gene panel. However, the observation of subpopulations expressing elevated levels of transcripts for most genes is not gene panel dependent. Our results are in agreement with earlier observations that transcription occurs in bursts (Raj et al., 2006; Sanchez and Golding, 2013), generating skewed distributions of transcripts among individual cells (Bengtsson et al., 2005).

In many organisms cell size is strongly correlated to cell division and growth rate (Dungrawala et al., 2010; Marguerat and Bahler, 2012), but the role of cell size in mammalian cells is less clear (Echave et al., 2007; Tzur et al., 2009). Our cell size data are in line with these reports. We observed increased numbers of small cells in the G1 phase using fluorescence activated cell sorting (Figure S1), but no clear correlation between cell size and total transcript levels were observed in any cell line. In MSC, we identified a subpopulation of small S and G2/M phase cells with distinct gene expression profile. The divergent results of MSC could be connected to the larger span in size variation of these cells compared to the other two cell lines (Figure 1A and Figure S1).

A large number of genes displayed correlations between their expression levels and cell cycle phase, while the number of correlations between expression level and cell size was fewer (Table 1 and Table S2). However, even for the genes with highest correlations we observed large overlap in gene expression levels among individual cells of different cell cycle phases and cell sizes (Figures 2C, 3C, 4C and Table S2). To further analyze the relations between gene expression and cell cycle phase respective cell size we applied the supervised random forests learning algorithm. This strategy generated a multi-gene signature that optimally separated pre-defined cell populations. Further, to identify the most predictive genes we applied RFE. Most of the predictive genes were similar in MLS 402-91 and MCF7, while MSC displayed a different gene list. Some genes, including *CCNB1* and *MKI67*, were predictive in all three cell lines. The RFE results showed that none of the measured genes alone or in combination could predict all cells into correct cell cycle phase or cell size in any cell line.

By excluding non-informative genes in the PCA we identified distinct G1′ subpopulations in both MLS 402-91 and MCF7. The G1′ subpopulations were characterized by low total transcript levels and downregulation of several proliferation associated genes. We speculate that these G1 phase cells are cells that have recently divided (Martinsson et al., 2005). One gene, *MCM6*, was upregulated in MLS 402-91, while downregulated in MCF7. *MCM6* belongs to the *MCM* gene family, where the MCM complex is loaded on chromatin exclusively during the G1 phase with help of other proteins, including CDT1 and CDC6 (Shetty et al., 2005). Interestingly, the second most upregulated gene in the MLS 402-91 G1′ subpopulation was *CDT1*, further indicating that the MCM complex may be differently regulated in MLS 402-91 compared to MCF7. The heterogeneously *MCM6* expression also translated into variable protein expression levels. Transcript data suggest that the cells with high MCM6 protein level in MLS 402-91 correspond to the G1′ subpopulation, while the opposite seems true for MCF7. Further, analyses are needed to define the cell line specific regulation of *MCM* genes.

A single parameter is easier to visualize and interpret than a multi-gene signature. Hence, we developed a cell cycle index to illustrate cell cycle progression. The index shows that cells are in continuous transition throughout the cell cycle until mitosis. In MLS 402-91 and MCF7 we observed a distinct cell cycle index crossover point for cells that were in the G1 to S phase transition (Figures 6A–B). We speculate that this cell cycle index breakpoint is related to the G1 restriction check point (Lubischer, 2007). The identified G1′ subpopulations in MLS 402-91 and MCF7 were characterized by low indexes, illustrating that these cells are not likely to enter the S phase in the near future. However, further analysis of more cell lines in different conditions, degree of differentiation and various genetic backgrounds is needed to determine general cell proliferation constraints. In addition, whole transcriptome analysis would most likely reveal more predictive genes allowing for a more detailed understanding of cell transitions between cell cycle phases.

## Author contributions

AS conceived and designed the study. AS, SD, NA, CV, TT performed the experiments. AS, JC, WL performed data analysis. All authors were involved in data interpretation and manuscript drafting. All authors approved the final manuscript.

## Funding

Barncancerfonden, BioCARE, Cancerfonden, Johan Jansson Stiftelsen för tumörforskning och cancerskadade, Sahlgrenska Akademin-ALF, Stiftelsen Assar Gabrielssons Fond, Stiftelserna Wilhelm och Martina Lundgrens Vetenskapsfond, VINNOVA, Åke Wiberg Stiftelse.

### Conflict of interest statement

The authors declare that the research was conducted in the absence of any commercial or financial relationships that could be construed as a potential conflict of interest.
