# Supplementary material for: Cell Cycle and Cell Size Dependent Gene Expression Reveals Distinct Subpopulations at Single-Cell Level
Source: Front Genet. 2017 Jan 25;8:1. doi: 10.3389/fgene.2017.00001 (PMC5263129; doi:10.3389/fgene.2017.00001)
Supplement: Supplementary file 3 [file Image1.PDF]

Cell cycle and cell size dependent gene expression reveals distinct subpopulations at single-cell level

Soheila Dolatabadi, Julián Candia, Nina Akrap, Christoffer Vannas, Tajana Tesan Tomic, Wolfgang Losert, Göran Landberg, Pierre Åman, Anders Ståhlberg

Supplementary material

Figure S1

Figure S2

Figure S3

Figure S4

Figure S5

Figure S6

Figure S7

Figure S8

Figure S9

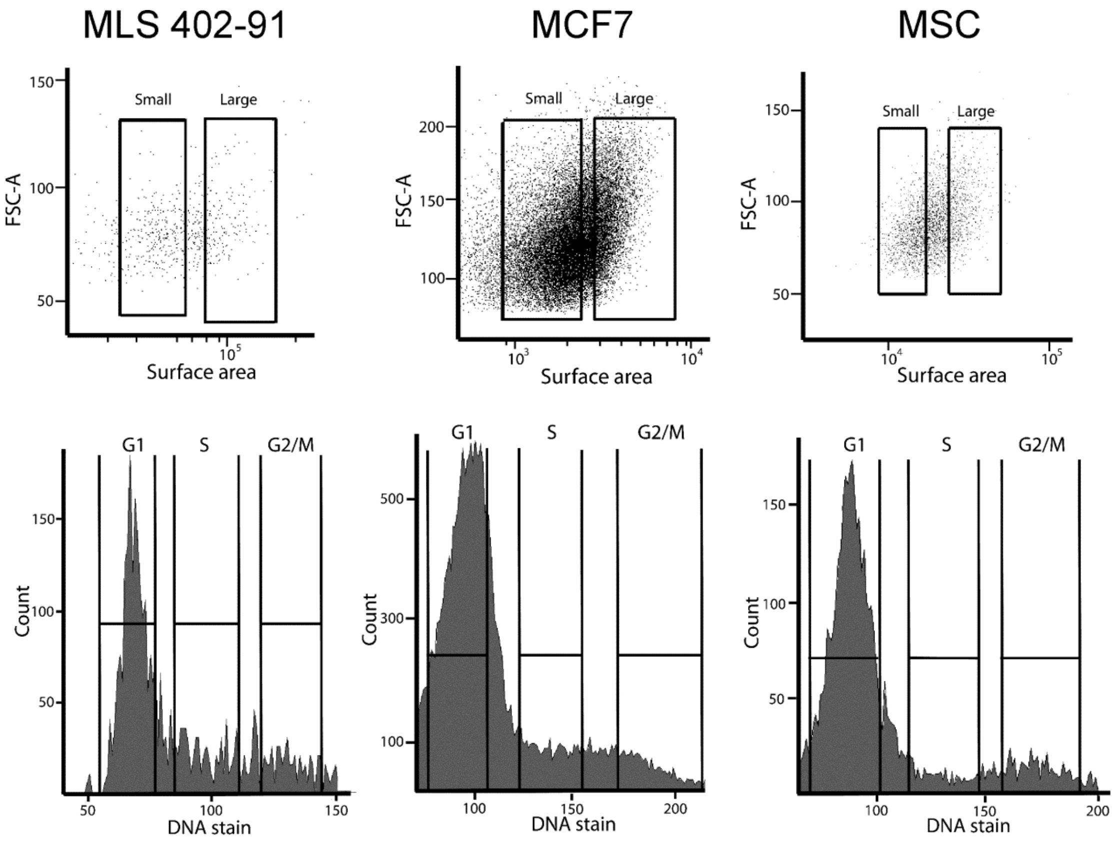

			Surface area (CellVue Claret)	DNA stain (Vybrant DyeCycle)	No. of cells (%)
MLS 402-91	Small	G1	52500	68.1	28.4
		S	52600	97.2	7.1
		G2/M	53700	131.6	4.1
	Large	G1	96400	69.8	8.0
		S	105000	96.8	5.0
		G2/M	110000	132.3	4.4
MCF7	Small	G1	1630	101	29.8
		S	1670	140	2.8
		G2/M	1710	175	1.3
	Large	G1	2880	98	18.1
		S	3170	137	6.1
		G2/M	3430	173	4.3
MSC	Small	G1	850	91.6	26.8
		S	820	137	2.9
		G2/M	890	181	3.0
	Large	G1	2180	94.8	16.5
		S	2410	128	2.1
		G2/M	2430	189	3.5

Figure S1. Collection of individual cells based on cell cycle phase and cell size using fluorescence activated cell sorting. Gate settings for different cell sizes and cell cycle phases are shown for MLS 402-91, MC7 and MSC. The table summarizes sorting statistics. Mean values of surface area and DNA stain are shown, as well as number of cells related to the whole ungated cell population.

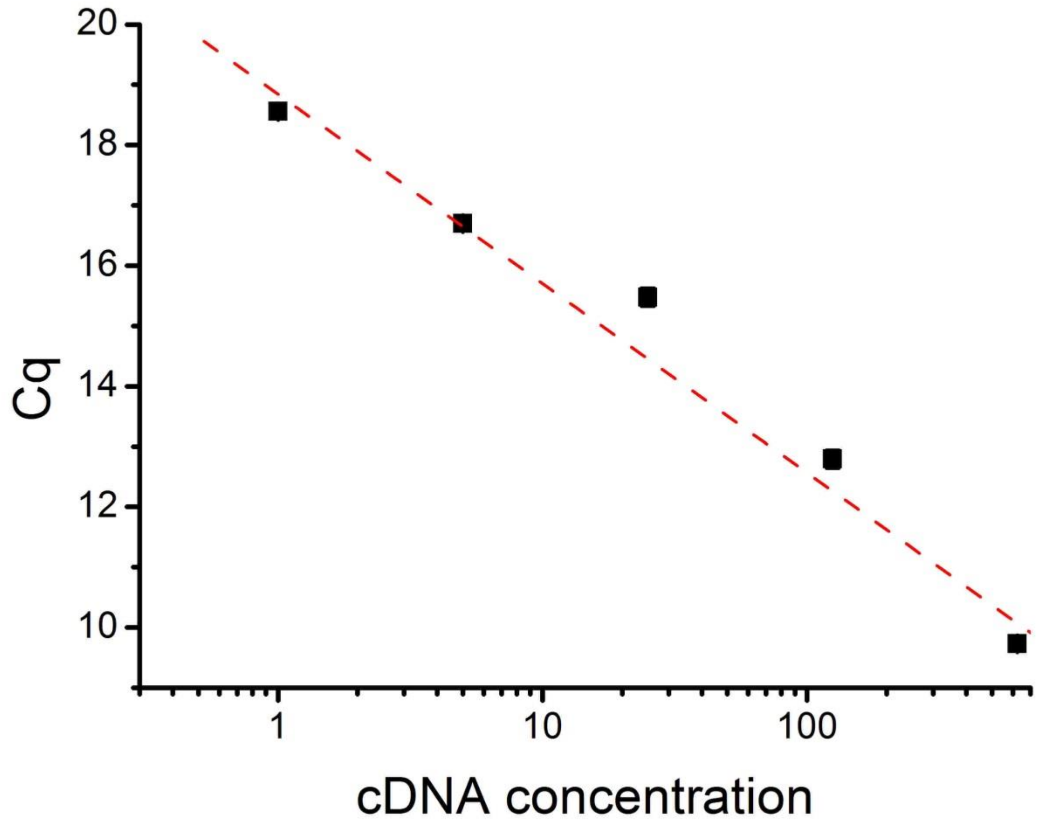

Figure S2. Preamplification performance. Preamplification of five different concentrations of cDNA (n = 4) were analyzed using BioMark real-time PCR system. Relative cDNA levels are shown where the lowest applied cDNA concentration was arbitrarily set to a value of one. Mean cycle of quantification (Cq) ± SD are plotted (n = 4). Each Cq-value was calculated as the average of all genes that were expressed in 4 or more dilutions. The overall PCR assay efficiency was 114 ± 15%.

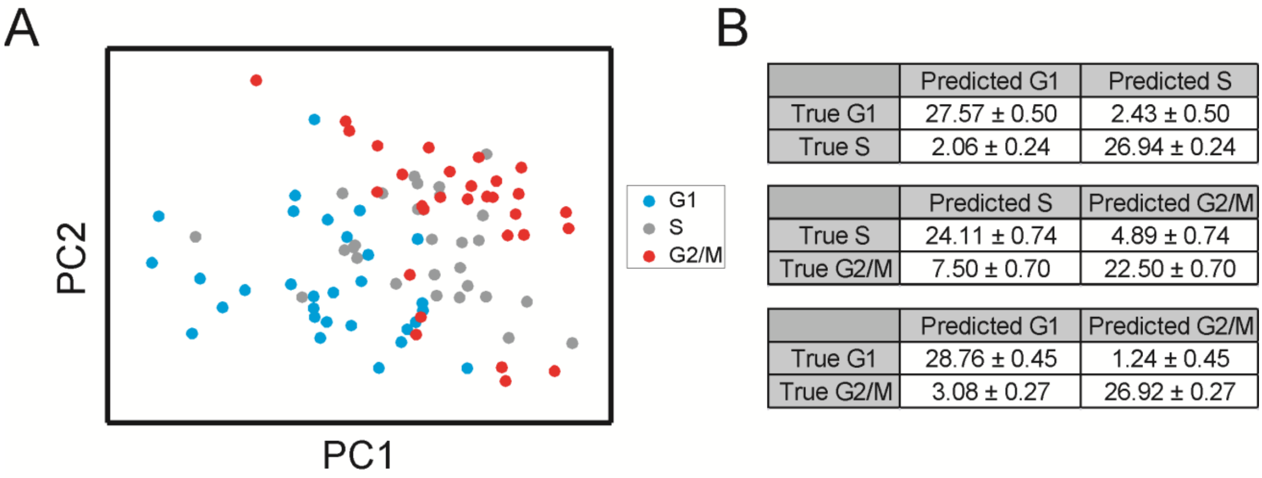

B

	Predicted G1	Predicted S
True G1	27.57 ± 0.50	2.43 ± 0.50
True S	2.06 ± 0.24	26.94 ± 0.24

	Predicted S	Predicted G2/M
True S	24.11 ± 0.74	4.89 ± 0.74
True G2/M	7.50 ± 0.70	22.50 ± 0.70

	Predicted G1	Predicted G2/M
True G1	28.76 ± 0.45	1.24 ± 0.45
True G2/M	3.08 ± 0.27	26.92 ± 0.27

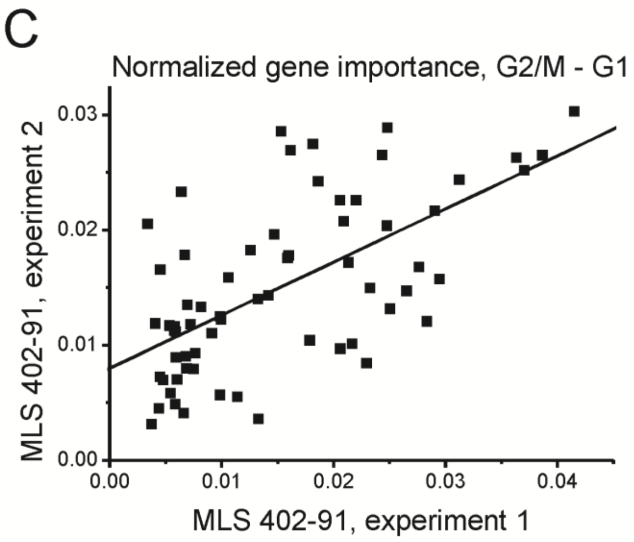

Figure S3. Principal component analysis and cell classification in a second MLS 402-91 data set. (A) PCA of MLS 402-91 cells in the G1, S and G2/M phases. Each dot represents a single cell. **(B)** Confusion matrices of cell classifications using the random forests algorithm. Fisher's exact test was used to calculate significance ($p < 0.0001$) for all matrices. **(C)** Correlation of gene importance identified by random forests algorithm between the two MLS 402-91 cell experiments. The normalized gene importance between G1 and G2/M cell cycle phases is shown for all genes. The best linear fit between the two data sets is shown ($R = 0.63$, $p < 0.0001$).

Figure S4.

MLS 402-91

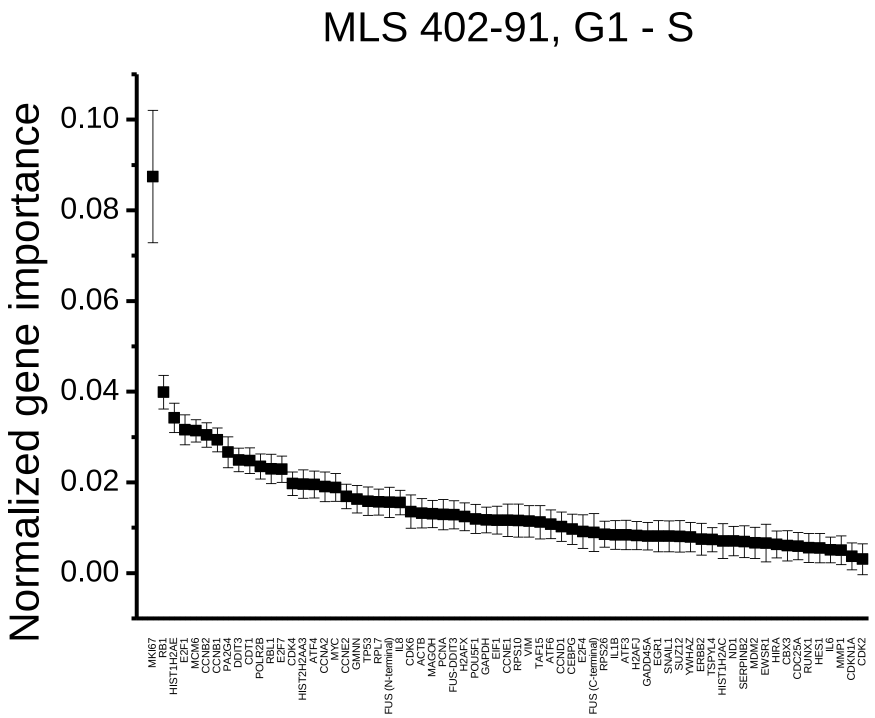

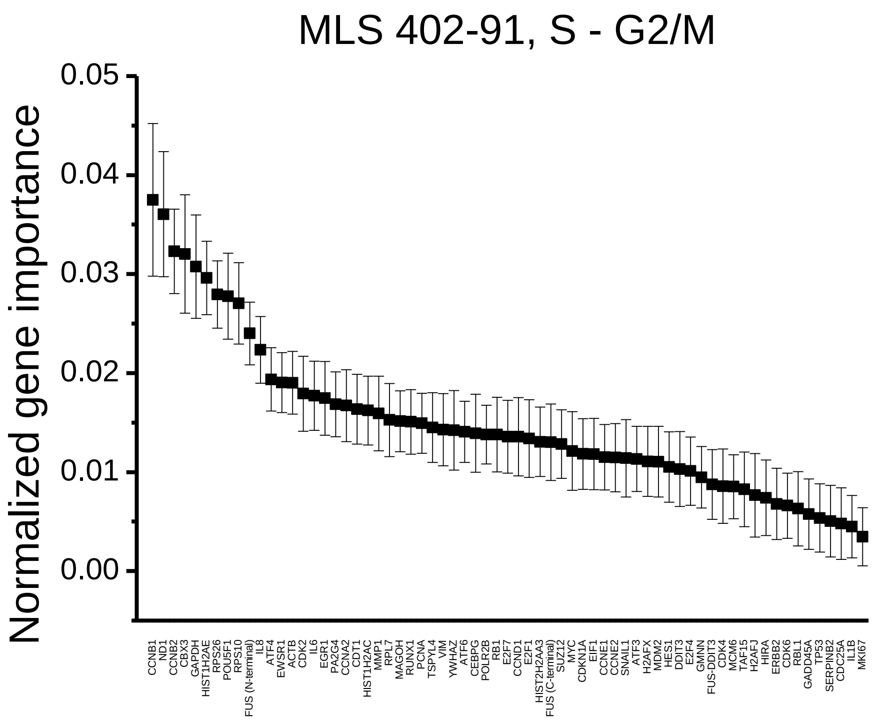
MLS 402-91, S - G2/M
Normalized gene importance
0.05
0.04
0.03
0.02
0.01
0.00
CCNB1
ND1
CCNB2
CBX3
GAPDH
HIST1H2AE
RPS26
POU5F1
RPS10
FUS (N-terminal)
IL8
ATF4
EWSR1
ACTB
CDK2
IL6
EGR1
PA2G4
CCNA2
CDT1
HIST1H2AC
MMP1
RPL7
MAGOH
RUNX1
PCNA
TSPYL4
VIM
YWHAZ
ATF6
CEBPG
POLR2B
RB1
E2F7
CCND1
E2F1
HIST2H2AA3
FUS (C-terminal)
SUZ12
MYC
CDKN1A
EIF1
CCNE1
CCNE2
SNAIL1
ATF3
H2AFX
MDM2
HES1
DDIT3
E2F4
GMNN
FUS-DDIT3
CDK4
MCM6
TAF15
H2AFJ
HIRA
ERBB2
CDK6
RBL1
GADD45A
TP53
SERPINB2
CDC25A
IL1B
MKI67

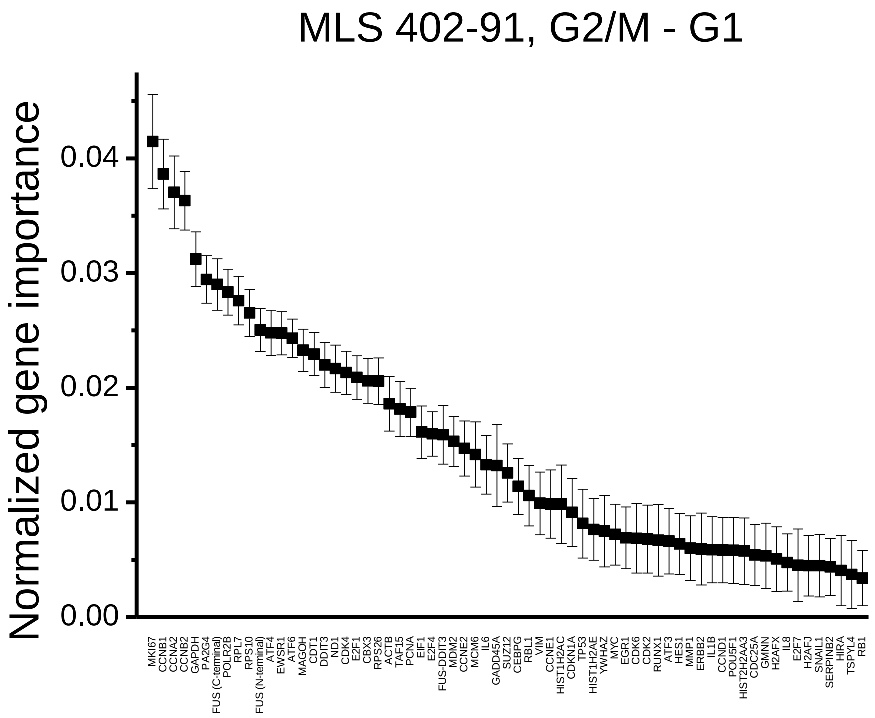

MLS 402-91, G2/M - G1
Normalized gene importance
0.00
0.01
0.02
0.03
0.04
MKI67
CCNB1
CCNA2
CCNB2
GAPDH
PA2G4
FUS (C-terminal)
POLR2B
RPL7
RPS10
FUS (N-terminal)
ATF4
EWSR1
ATF6
MAGOH
CDT1
DDIT3
ND1
CDK4
E2F1
CBX3
RPS26
ACTB
TAF15
PCNA
EIF1
E2F4
FUS-DDIT3
MDM2
CCNE2
MCM6
IL6
GADD45A
SUZ12
CEBPG
RBL1
VIM
CCNE1
HIST1H2AC
CDKN1A
TP53
HIST1H2AE
YWHAZ
MYC
EGR1
CDK6
CDK2
RUNX1
ATF3
HES1
MMP1
ERBB2
IL1B
CCND1
POU5F1
HIST2H2AA3
CDC25A
GMNN
H2AFX
IL8
E2F7
H2AFJ
SNAIL1
SERPINB2
HIRA
TSPYL4
RB1

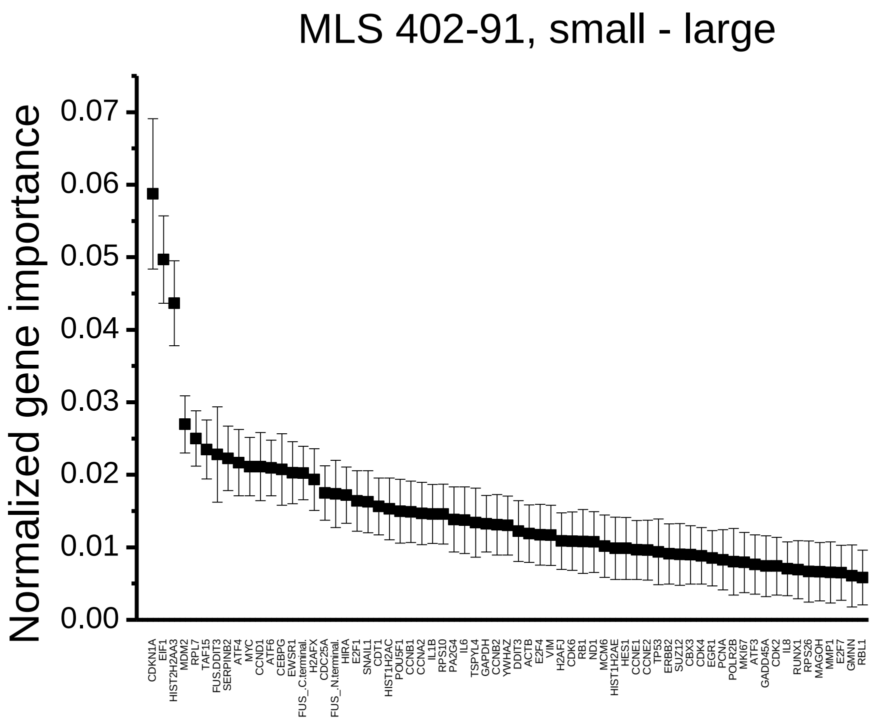
MLS 402-91, small - large
Normalized gene importance
0.00
0.01
0.02
0.03
0.04
0.05
0.06
0.07
CDKN1A
EIF1
HIST2H2AA3
MDM2
RPL7
TAF15
FUS.DDIT3
SERPINB2
ATF4
MYC
CCND1
ATF6
CEBPG
EWSR1
FUS_.C.terminal.
H2AFX
CDC25A
FUS_.N.terminal.
HIRA
E2F1
SNAIL1
CDT1
HIST1H2AC
POU5F1
CCNB1
CCNA2
IL1B
RPS10
PA2G4
IL6
TSPYL4
GAPDH
CCNB2
YWHAZ
DDIT3
ACTB
E2F4
VIM
H2AFJ
CDK6
RB1
ND1
MCM6
HIST1H2AE
HES1
CCNE1
CCNE2
TP53
ERBB2
SUZ12
CBX3
CDK4
EGR1
PCNA
POLR2B
MKI67
ATF3
GADD45A
CDK2
IL8
RUNX1
RPS26
MAGOH
MMP1
E2F7
GMNN
RBL1

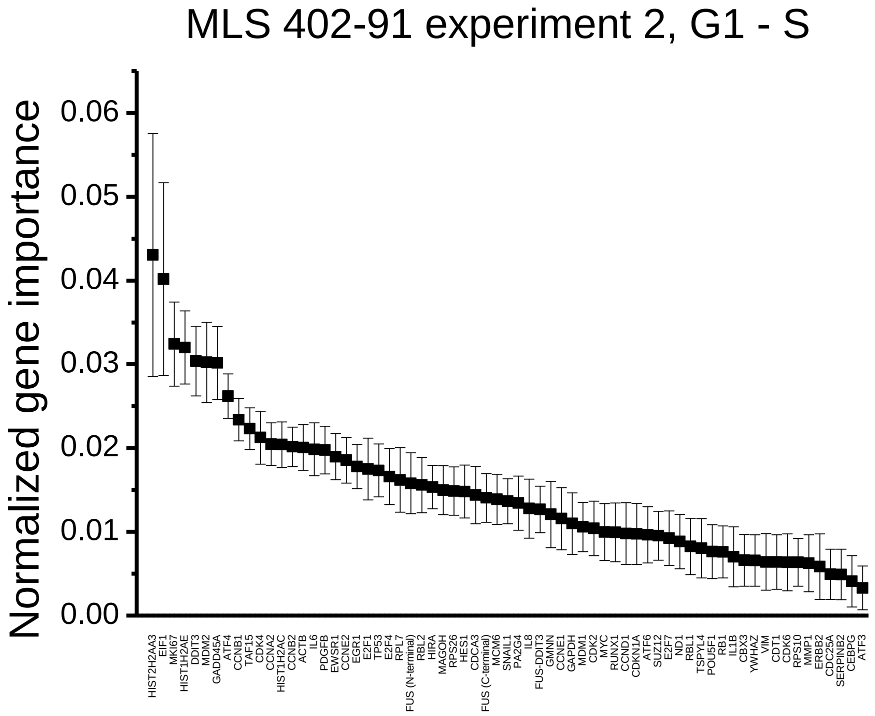
MLS 402-91 experiment 2, G1 - S
Normalized gene importance
0.06
0.05
0.04
0.03
0.02
0.01
0.00
HIST2H2AA3
EIF1
MKI67
HIST1H2AE
DDIT3
MDM2
GADD45A
ATF4
CCNB1
TAF15
CDK4
CCNA2
HIST1H2AC
CCNB2
ACTB
IL6
PDGFB
EWSR1
CCNE2
EGR1
E2F1
TP53
E2F4
RPL7
FUS (N-terminal)
RBL2
HIRA
MAGOH
RPS26
HES1
CDCA3
FUS (C-terminal)
MCM6
SNAIL1
PA2G4
IL8
FUS-DDIT3
GMNN
CCNE1
GAPDH
MDM1
CDK2
MYC
RUNX1
CCND1
CDKN1A
ATF6
SUZ12
E2F7
ND1
RBL1
TSPYL4
POU5F1
RB1
IL1B
CBX3
YWHAZ
VIM
CDT1
CDK6
RPS10
MMP1
ERBB2
CDC25A
SERPINB2
CEBPG
ATF3

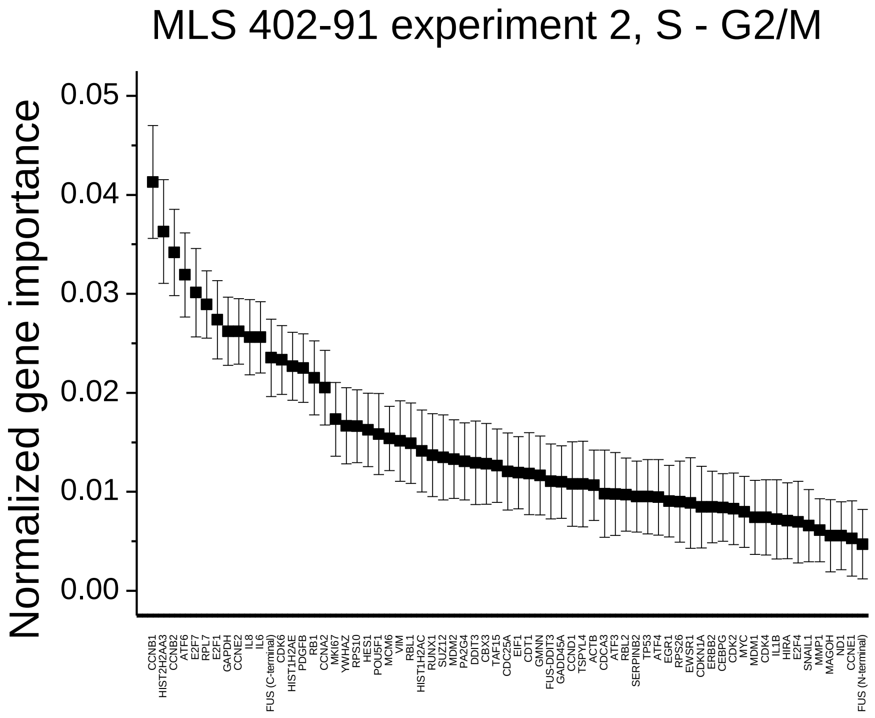
MLS 402-91 experiment 2, S - G2/M
Normalized gene importance
0.05
0.04
0.03
0.02
0.01
0.00
CCNB1
HIST2H2AA3
CCNB2
ATF6
E2F7
RPL7
E2F1
GAPDH
CCNE2
IL8
IL6
FUS (C-terminal)
CDK6
HIST1H2AE
PDGFB
RB1
CCNA2
MKI67
YWHAZ
RPS10
HES1
POU5F1
MCM6
VIM
RBL1
HIST1H2AC
RUNX1
SUZ12
MDM2
PA2G4
DDIT3
CBX3
TAF15
CDC25A
EIF1
CDT1
GMNN
FUS-DDIT3
GADD45A
CCND1
TSPYL4
ACTB
CDCA3
ATF3
RBL2
SERPINB2
TP53
ATF4
EGR1
RPS26
EWSR1
CDKN1A
ERBB2
CEBPG
CDK2
MYC
MDM1
CDK4
IL1B
HIRA
E2F4
SNAIL1
MMP1
MAGOH
ND1
CCNE1
FUS (N-terminal)

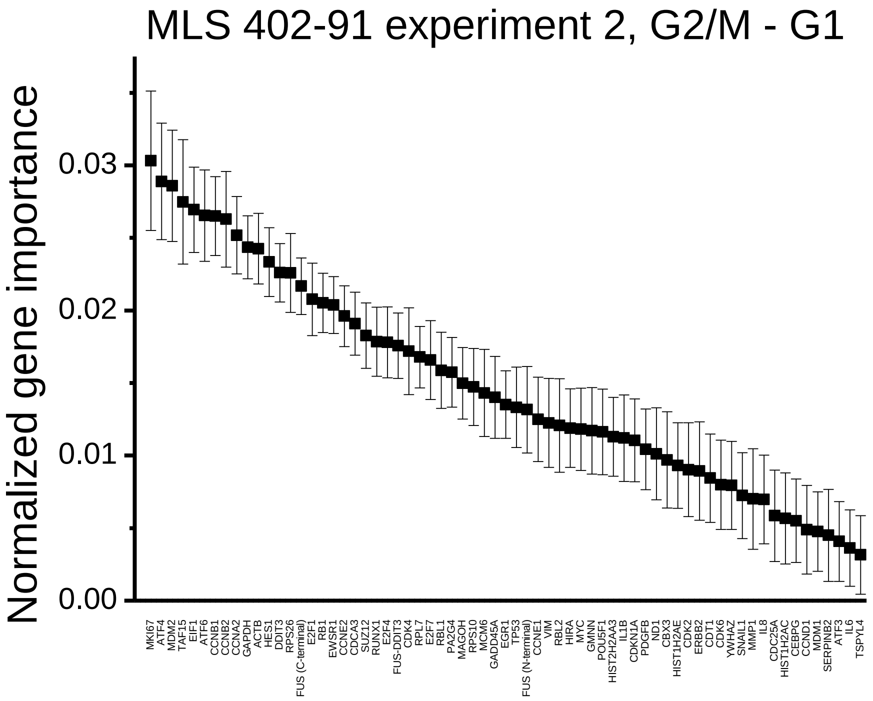
MLS 402-91 experiment 2, G2/M - G1
Normalized gene importance
0.03
0.02
0.01
0.00
MKI67
ATF4
MDM2
TAF15
EIF1
ATF6
CCNB1
CCNB2
CCNA2
GAPDH
ACTB
HES1
DDIT3
RPS26
FUS (C-terminal)
E2F1
RB1
EWSR1
CCNE2
CDCA3
SUZ12
RUNX1
E2F4
FUS-DDIT3
CDK4
RPL7
E2F7
RBL1
PA2G4
MAGOH
RPS10
MCM6
GADD45A
EGR1
TP53
FUS (N-terminal)
CCNE1
VIM
RBL2
HIRA
MYC
GMNN
POU5F1
HIST2H2AA3
IL1B
CDKN1A
PDGFB
ND1
CBX3
HIST1H2AE
CDK2
ERBB2
CDT1
CDK6
YWHAZ
SNAIL1
MMP1
IL8
CDC25A
HIST1H2AC
CEBPG
CCND1
MDM1
SERPINB2
ATF3
IL6
TSPYL4

MCF7

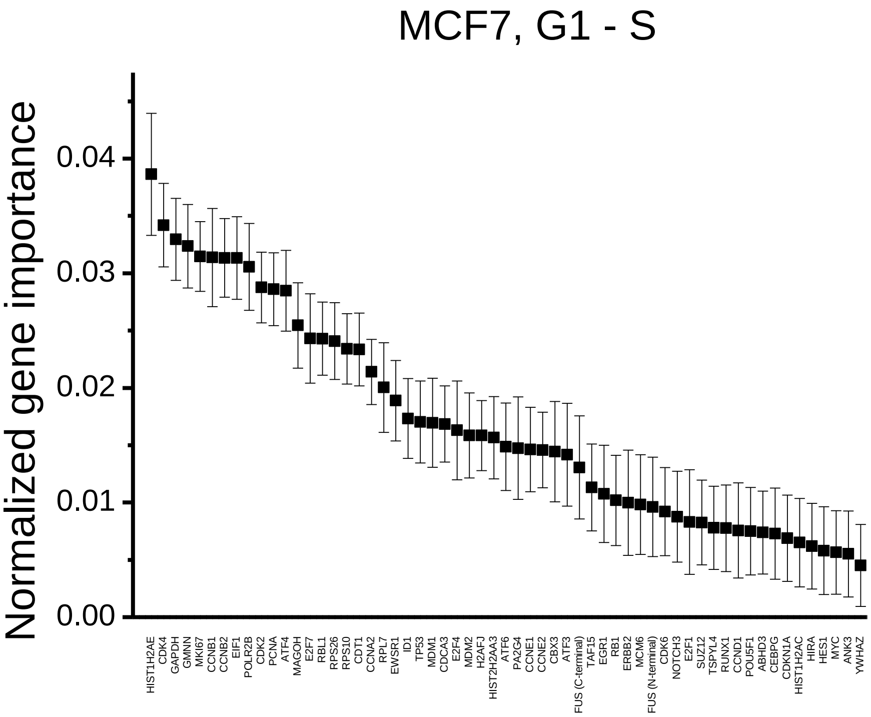
MCF7, G1 - S
Normalized gene importance
0.04
0.03
0.02
0.01
0.00
HIST1H2AE
CDK4
GAPDH
GMNN
MKI67
CCNB1
CCNB2
EIF1
POLR2B
CDK2
PCNA
ATF4
MAGOH
E2F7
RBL1
RPS26
RPS10
CDT1
CCNA2
RPL7
EWSR1
ID1
TP53
MDM1
CDCA3
E2F4
MDM2
H2AFJ
HIST2H2AA3
ATF6
PA2G4
CCNE1
CCNE2
CBX3
ATF3
FUS (C-terminal)
TAF15
EGR1
RB1
ERBB2
MCM6
FUS (N-terminal)
CDK6
NOTCH3
E2F1
SUZ12
TSPYL4
RUNX1
CCND1
POU5F1
ABHD3
CEBPG
CDKN1A
HIST1H2AC
HIRA
HES1
MYC
ANK3
YWHAZ

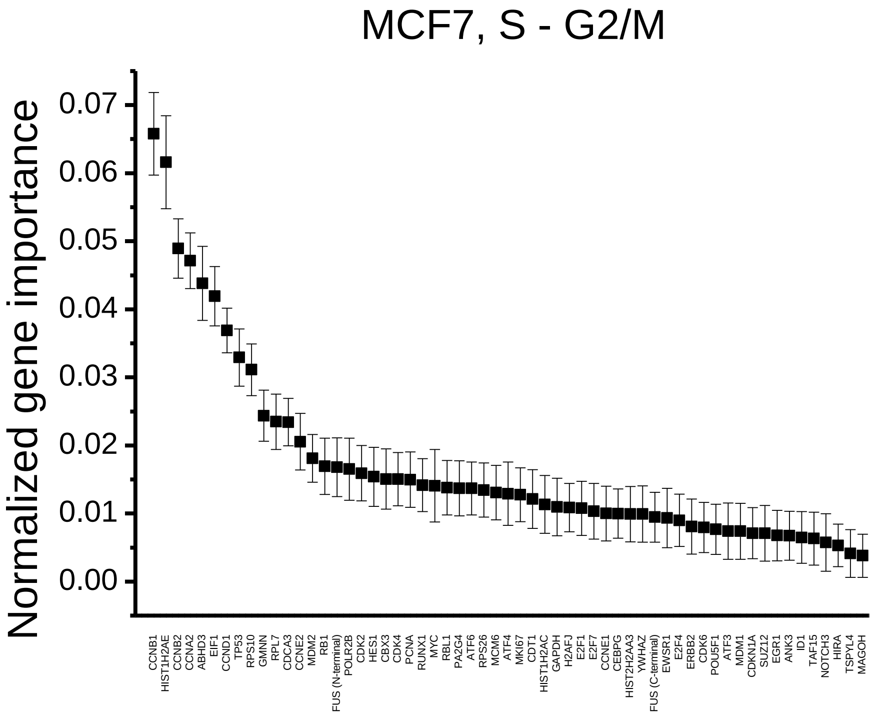
MCF7, S - G2/M
Normalized gene importance
0.07
0.06
0.05
0.04
0.03
0.02
0.01
0.00
CCNB1
HIST1H2AE
CCNB2
CCNA2
ABHD3
EIF1
CCND1
TP53
RPS10
GMNN
RPL7
CDCA3
CCNE2
MDM2
RB1
FUS (N-terminal)
POLR2B
CDK2
HES1
CBX3
CDK4
PCNA
RUNX1
MYC
RBL1
PA2G4
ATF6
RPS26
MCM6
ATF4
MKI67
CDT1
HIST1H2AC
GAPDH
H2AFJ
E2F1
E2F7
CCNE1
CEBPG
HIST2H2AA3
YWHAZ
FUS (C-terminal)
EWSR1
E2F4
ERBB2
CDK6
POU5F1
ATF3
MDM1
CDKN1A
SUZ12
EGR1
ANK3
ID1
TAF15
NOTCH3
HIRA
TSPYL4
MAGOH

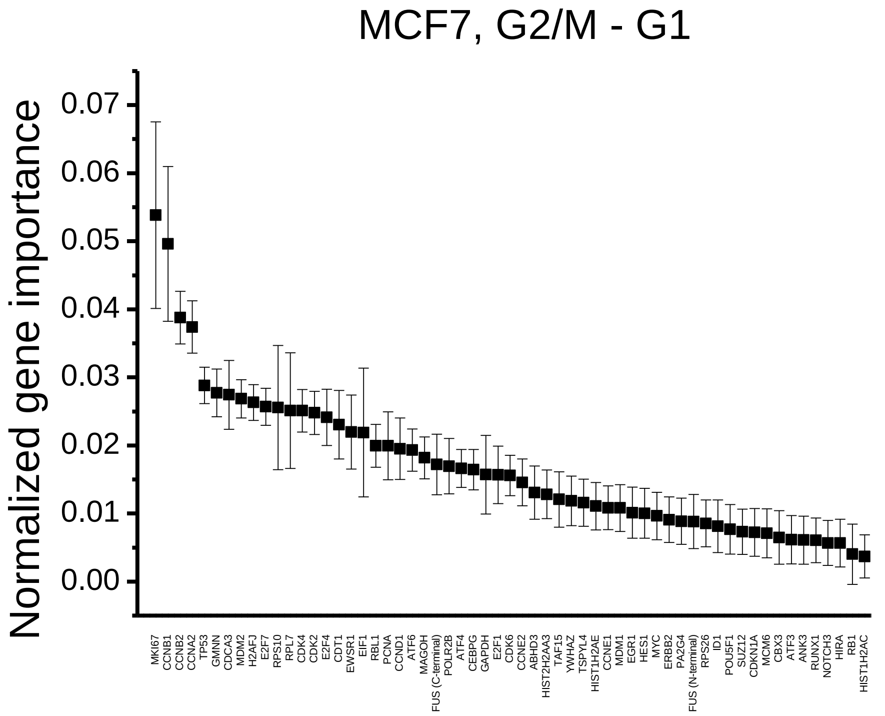
MCF7, G2/M - G1
Normalized gene importance
0.07
0.06
0.05
0.04
0.03
0.02
0.01
0.00
MKI67
CCNB1
CCNB2
CCNA2
TP53
GMNN
CDCA3
MDM2
H2AFJ
E2F7
RPS10
RPL7
CDK4
CDK2
E2F4
CDT1
EWSR1
EIF1
RBL1
PCNA
CCND1
ATF6
MAGOH
FUS (C-terminal)
POLR2B
ATF4
CEBPG
GAPDH
E2F1
CDK6
CCNE2
ABHD3
HIST2H2AA3
TAF15
YWHAZ
TSPYL4
HIST1H2AE
CCNE1
MDM1
EGR1
HES1
MYC
ERBB2
PA2G4
FUS (N-terminal)
RPS26
ID1
POU5F1
SUZ12
CDKN1A
MCM6
CBX3
ATF3
ANK3
RUNX1
NOTCH3
HIRA
RB1
HIST1H2AC

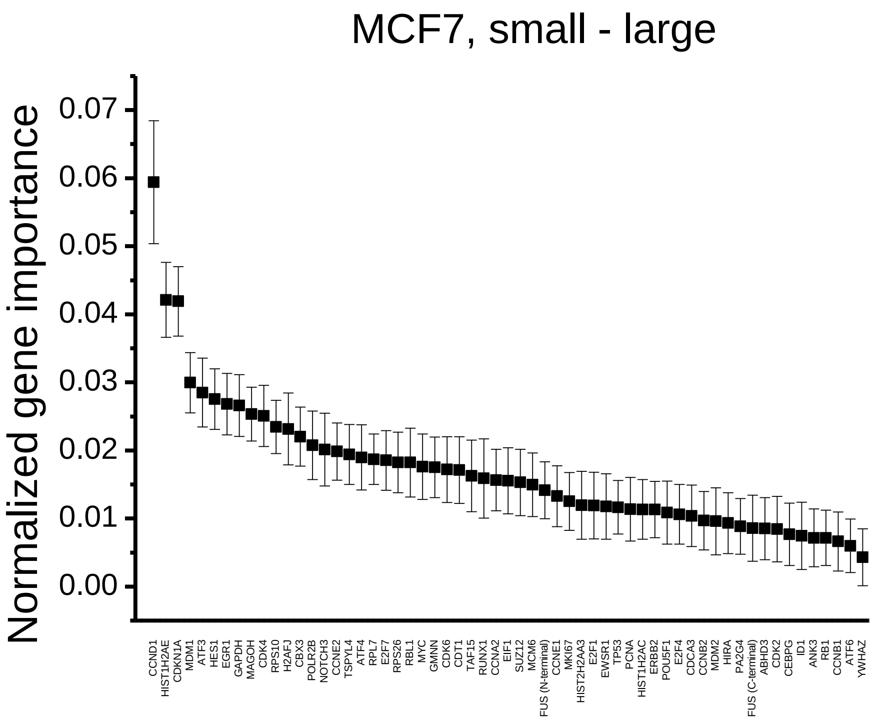
MCF7, small - large
Normalized gene importance
0.07
0.06
0.05
0.04
0.03
0.02
0.01
0.00
CCND1
HIST1H2AE
CDKN1A
MDM1
ATF3
HES1
EGR1
GAPDH
MAGOH
CDK4
RPS10
H2AFJ
CBX3
POLR2B
NOTCH3
CCNE2
TSPYL4
ATF4
RPL7
E2F7
RPS26
RBL1
MYC
GMNN
CDK6
CDT1
TAF15
RUNX1
CCNA2
EIF1
SUZ12
MCM6
FUS (N-terminal)
CCNE1
MKI67
HIST2H2AA3
E2F1
EWSR1
TP53
PCNA
HIST1H2AC
ERBB2
POU5F1
E2F4
CDCA3
CCNB2
MDM2
HIRA
PA2G4
FUS (C-terminal)
ABHD3
CDK2
CEBPG
ID1
ANK3
RB1
CCNB1
ATF6
YWHAZ

MSC

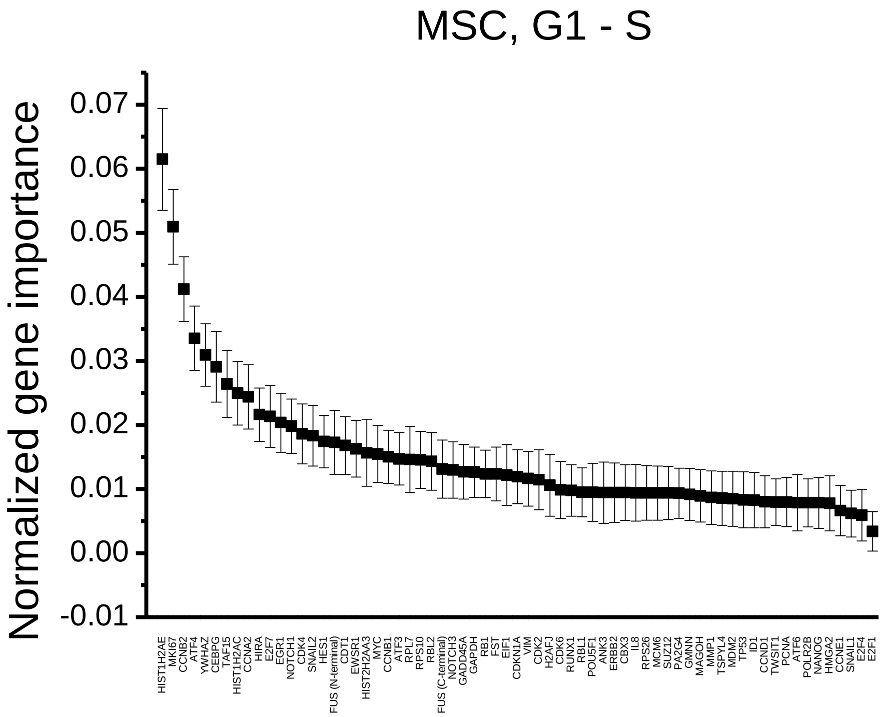
MSC, G1 - S
Normalized gene importance
0.07
0.06
0.05
0.04
0.03
0.02
0.01
0.00
-0.01
HIST1H2AE
MKI67
CCNB2
ATF4
YWHAZ
CEBPG
TAF15
HIST1H2AC
CCNA2
HIRA
E2F7
EGR1
NOTCH1
CDK4
SNAIL2
HES1
FUS (N-terminal)
CDT1
EWSR1
HIST2H2AA3
MYC
CCNB1
ATF3
RPL7
RPS10
RBL2
FUS (C-terminal)
NOTCH3
GADD45A
GAPDH
RB1
FST
EIF1
CDKN1A
VIM
CDK2
H2AFJ
CDK6
RUNX1
RBL1
POU5F1
ANK3
ERBB2
CBX3
IL8
RPS26
MCM6
SUZ12
PA2G4
GMNN
MAGOH
MMP1
TSPYL4
MDM2
TP53
ID1
CCND1
TWSIT1
PCNA
ATF6
POLR2B
NANOG
HMGA2
CCNE1
SNAIL1
E2F4
E2F1

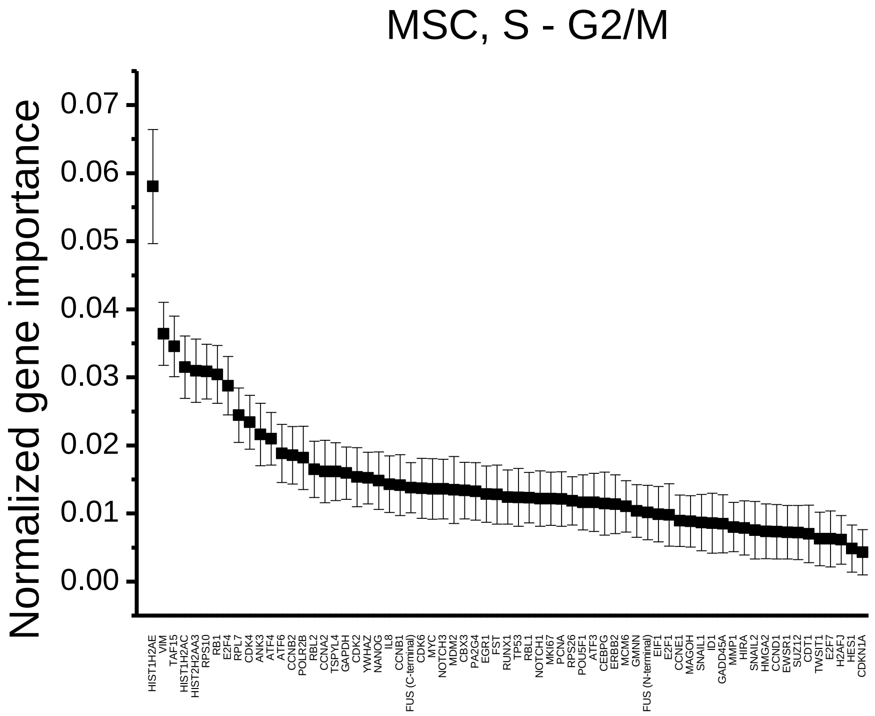
MSC, S - G2/M
Normalized gene importance
0.07
0.06
0.05
0.04
0.03
0.02
0.01
0.00
HIST1H2AE
VIM
TAF15
HIST1H2AC
HIST2H2AA3
RPS10
RB1
E2F4
RPL7
CDK4
ANK3
ATF4
ATF6
CCNB2
POLR2B
RBL2
CCNA2
TSPYL4
GAPDH
CDK2
YWHAZ
NANOG
IL8
CCNB1
FUS (C-terminal)
CDK6
MYC
NOTCH3
MDM2
CBX3
PA2G4
EGR1
FST
RUNX1
TP53
RBL1
NOTCH1
MKI67
PCNA
RPS26
POU5F1
ATF3
CEBPG
ERBB2
MCM6
GMNN
FUS (N-terminal)
EIF1
E2F1
CCNE1
MAGOH
SNAIL1
ID1
GADD45A
MMP1
HIRA
SNAIL2
HMGA2
CCND1
EWSR1
SUZ12
CDT1
TWSIT1
E2F7
H2AFJ
HES1
CDKN1A

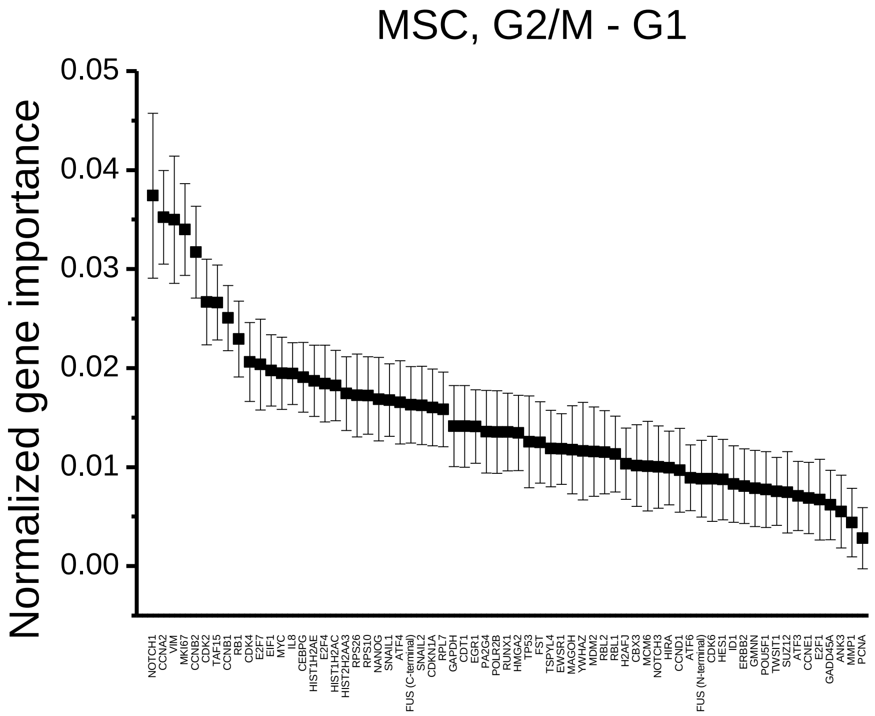
MSC, G2/M - G1
Normalized gene importance
0.05
0.04
0.03
0.02
0.01
0.00
NOTCH1
CCNA2
VIM
MKI67
CCNB2
CDK2
TAF15
CCNB1
RB1
CDK4
E2F7
EIF1
MYC
IL8
CEBPG
HIST1H2AE
E2F4
HIST1H2AC
HIST2H2AA3
RPS26
RPS10
NANOG
SNAIL1
ATF4
FUS (C-terminal)
SNAIL2
CDKN1A
RPL7
GAPDH
CDT1
EGR1
PA2G4
POLR2B
RUNX1
HMGA2
TP53
FST
TSPYL4
EWSR1
MAGOH
YWHAZ
MDM2
RBL2
RBL1
H2AFJ
CBX3
MCM6
NOTCH3
HIRA
CCND1
ATF6
FUS (N-terminal)
CDK6
HES1
ID1
ERBB2
GMNN
POU5F1
TWSIT1
SUZ12
ATF3
CCNE1
E2F1
GADD45A
ANK3
MMP1
PCNA

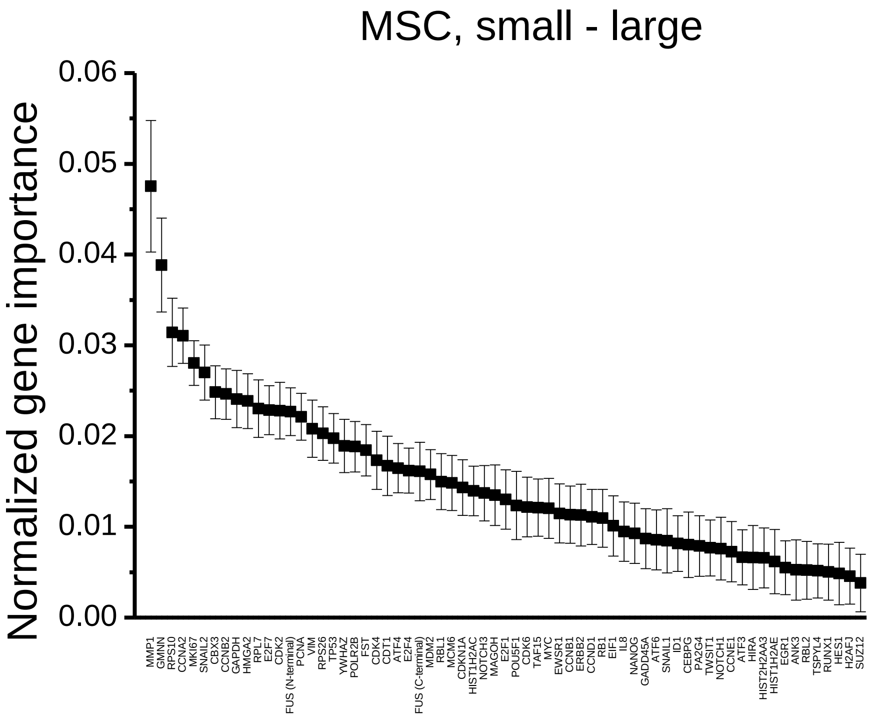

Figure S4. Normalized gene importance using the random forests algorithm. Two groups of cells were classified using the random forests algorithm. Small and large cells were analyzed together when classifying cells into their cell cycle phases. In the same way, G1, S and G2/M phase cells were analyzed together when classifying cells as small or large. For details, see Material and Methods. Mean ± SD is shown.

Figure S5.

MLS 402-91

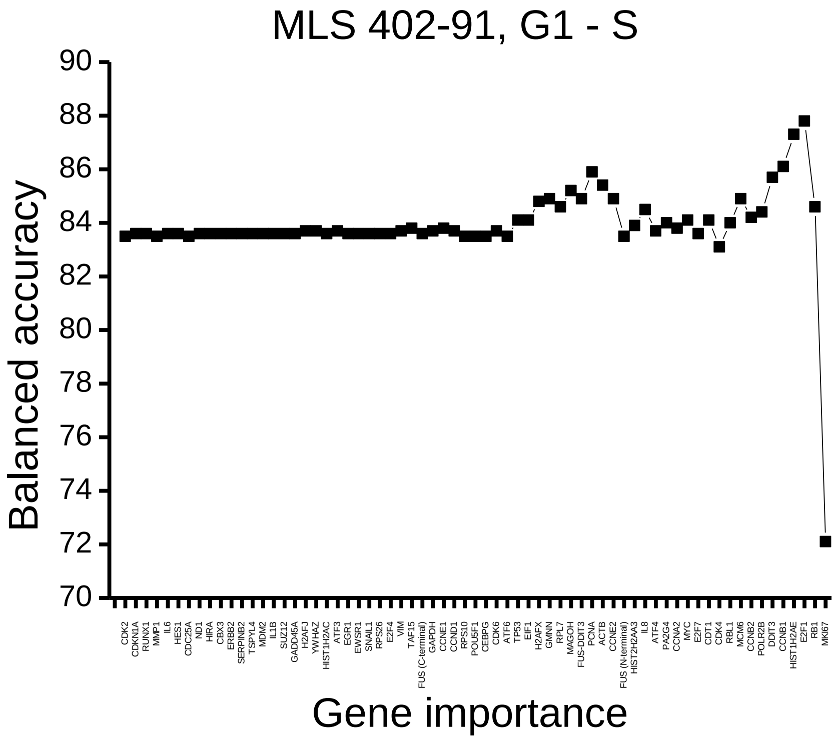

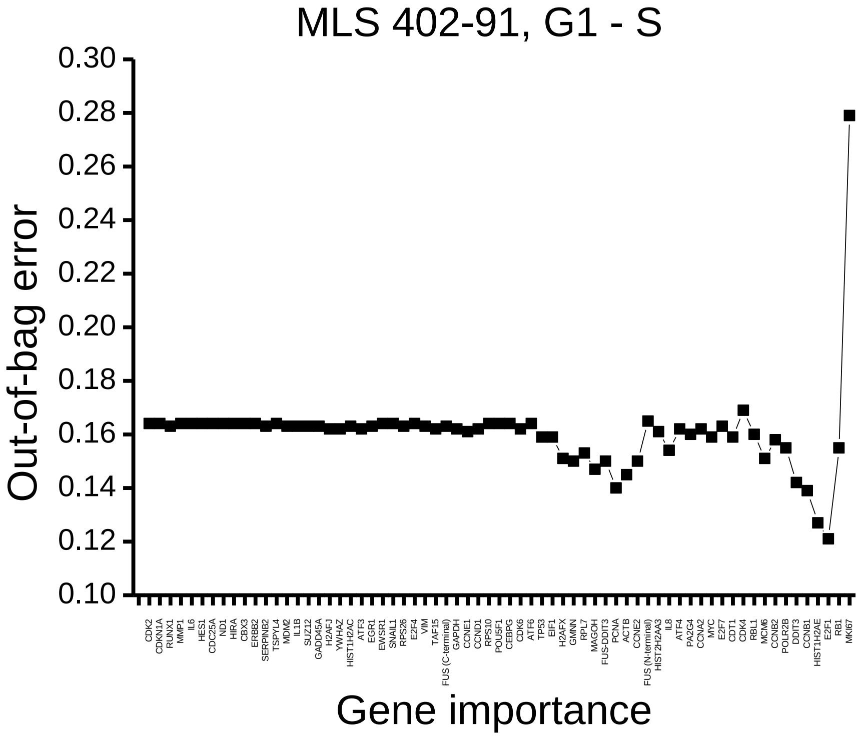
MLS 402-91, G1 - S
Out-of-bag error
0.30
0.28
0.26
0.24
0.22
0.20
0.18
0.16
0.14
0.12
0.10
CDK2
CDKN1A
RUNX1
MMP1
IL6
HES1
CDC25A
ND1
HIRA
CBX3
ERBB2
SERPINB2
TSPYL4
MDM2
IL1B
SUZ12
GADD45A
H2AFJ
YWHAZ
HIST1H2AC
ATF3
EGR1
EWSR1
SNAIL1
RPS26
E2F4
VIM
TAF15
FUS (C-terminal)
GAPDH
CCNE1
CCND1
RPS10
POU5F1
CEBPG
CDK6
ATF6
TP53
EIF1
H2AFX
GMNN
RPL7
MAGOH
FUS-DDIT3
PCNA
ACTB
CCNE2
FUS (N-terminal)
HIST2H2AA3
IL8
ATF4
PA2G4
CCNA2
MYC
E2F7
CDT1
CDK4
RBL1
MCM6
CCNB2
POLR2B
DDIT3
CCNB1
HIST1H2AE
E2F1
RB1
MKI67
Gene importance

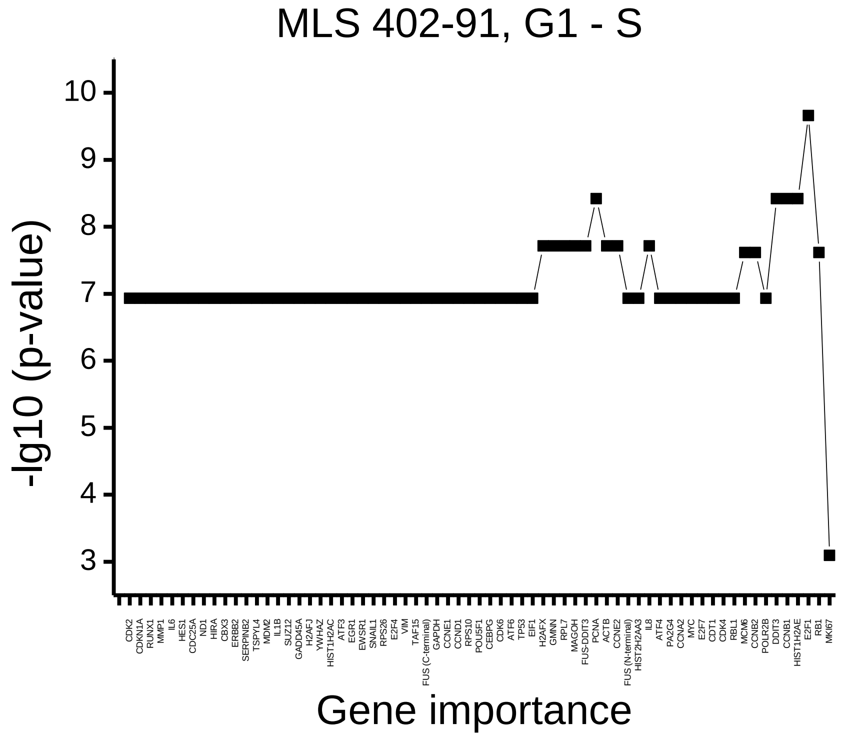
MLS 402-91, G1 - S
-lg10 (p-value)
10
9
8
7
6
5
4
3
CDK2
CDKN1A
RUNX1
MMP1
IL6
HES1
CDC25A
ND1
HIRA
CBX3
ERBB2
SERPINB2
TSPYL4
MDM2
IL1B
SUZ12
GADD45A
H2AFJ
YWHAZ
HIST1H2AC
ATF3
EGR1
EWSR1
SNAIL1
RPS26
E2F4
VIM
TAF15
FUS (C-terminal)
GAPDH
CCNE1
CCND1
RPS10
POU5F1
CEBPG
CDK6
ATF6
TP53
EIF1
H2AFX
GMNN
RPL7
MAGOH
FUS-DDIT3
PCNA
ACTB
CCNE2
FUS (N-terminal)
HIST2H2AA3
IL8
ATF4
PA2G4
CCNA2
MYC
E2F7
CDT1
CDK4
RBL1
MCM6
CCNB2
POLR2B
DDIT3
CCNB1
HIST1H2AE
E2F1
RB1
MKI67
Gene importance

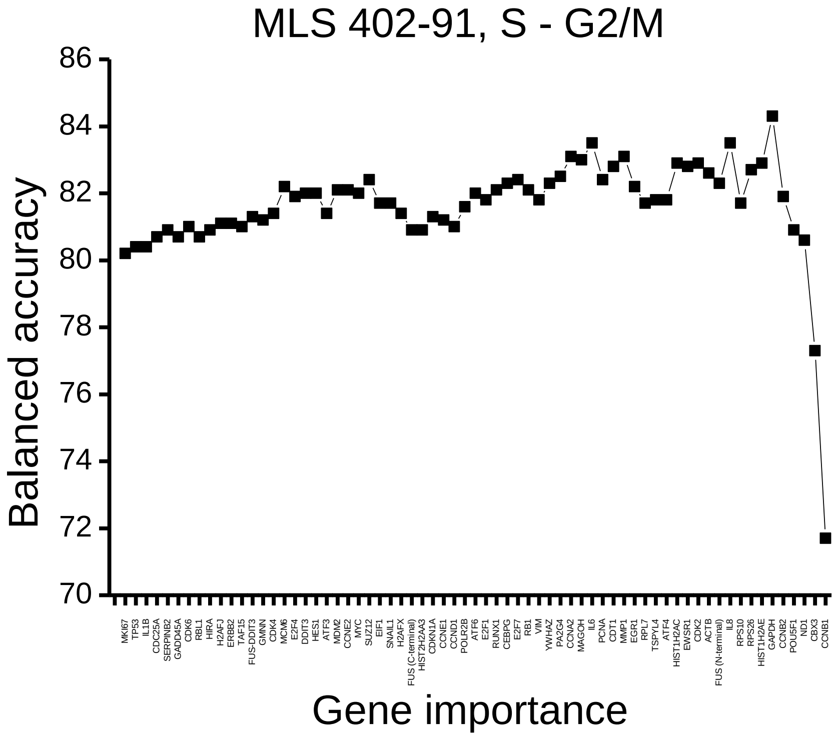
MLS 402-91, S - G2/M
Balanced accuracy
86
84
82
80
78
76
74
72
70
MKI67
TP53
IL1B
CDC25A
SERPINB2
GADD45A
CDK6
RBL1
HIRA
H2AFJ
ERBB2
TAF15
FUS-DDIT3
GMNN
CDK4
MCM6
E2F4
DDIT3
HES1
ATF3
MDM2
CCNE2
MYC
SUZ12
EIF1
SNAIL1
H2AFX
FUS (C-terminal)
HIST2H2AA3
CDKN1A
CCNE1
CCND1
POLR2B
ATF6
E2F1
RUNX1
CEBPG
E2F7
RB1
VIM
YWHAZ
PA2G4
CCNA2
MAGOH
IL6
PCNA
CDT1
MMP1
EGR1
RPL7
TSPYL4
ATF4
HIST1H2AC
EWSR1
CDK2
ACTB
FUS (N-terminal)
IL8
RPS10
RPS26
HIST1H2AE
GAPDH
CCNB2
POU5F1
ND1
CBX3
CCNB1
Gene importance

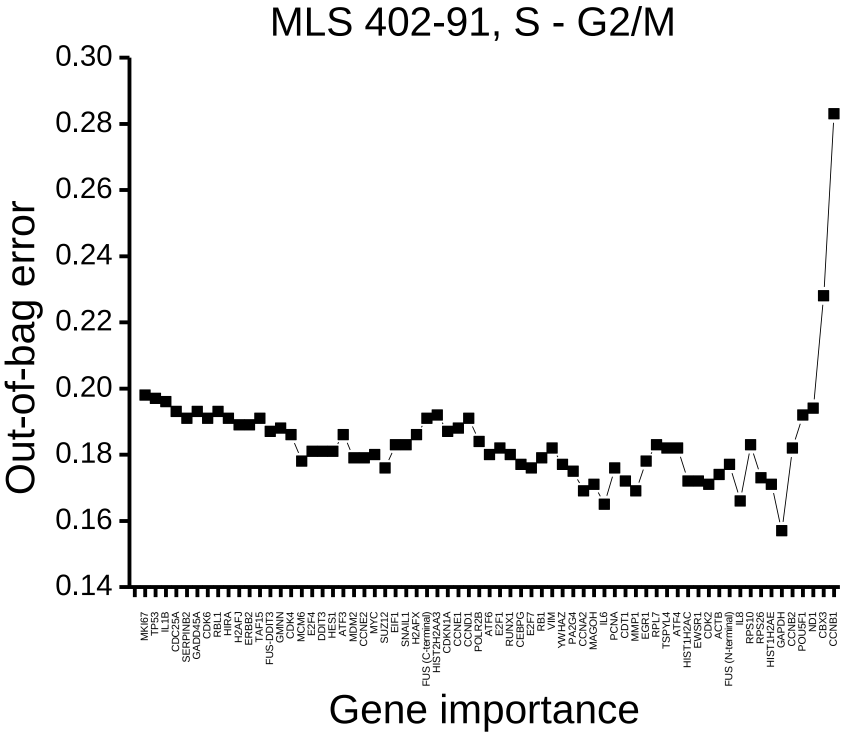
MLS 402-91, S - G2/M
Out-of-bag error
0.30
0.28
0.26
0.24
0.22
0.20
0.18
0.16
0.14
MKI67
TP53
IL1B
CDC25A
SERPINB2
GADD45A
CDK6
RBL1
HIRA
H2AFJ
ERBB2
TAF15
FUS-DDIT3
GMNN
CDK4
MCM6
E2F4
DDIT3
HES1
ATF3
MDM2
CCNE2
MYC
SUZ12
EIF1
SNAIL1
H2AFX
FUS (C-terminal)
HIST2H2AA3
CDKN1A
CCNE1
CCND1
POLR2B
ATF6
E2F1
RUNX1
CEBPG
E2F7
RB1
VIM
YWHAZ
PA2G4
CCNA2
MAGOH
IL6
PCNA
CDT1
MMP1
EGR1
RPL7
TSPYL4
ATF4
HIST1H2AC
EWSR1
CDK2
ACTB
FUS (N-terminal)
IL8
RPS10
RPS26
HIST1H2AE
GAPDH
CCNB2
POU5F1
ND1
CBX3
CCNB1
Gene importance

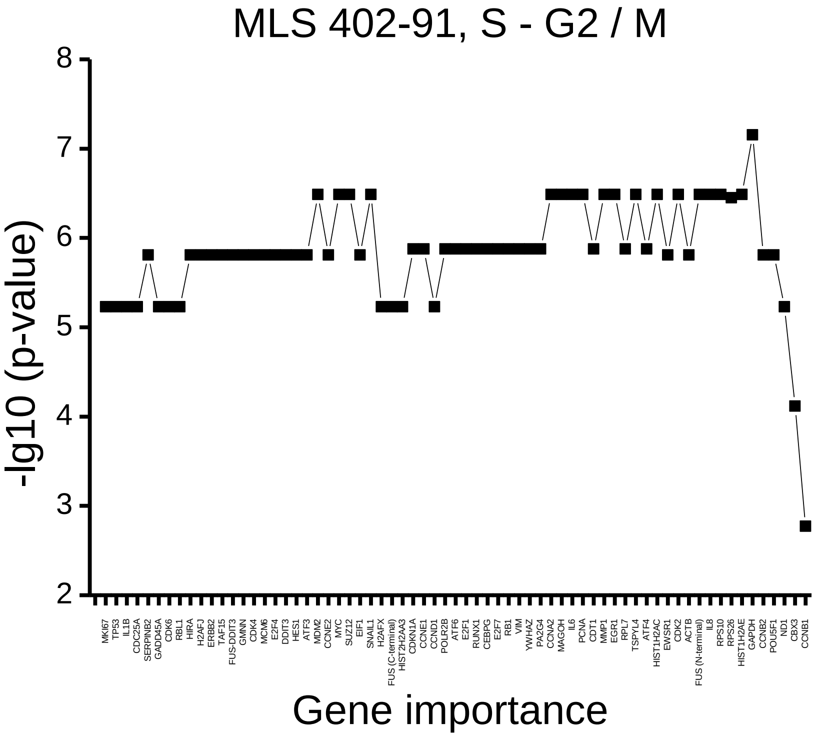

MLS 402-91, S - G2 / M
-lg10 (p-value)
8
7
6
5
4
3
2
MKI67
TP53
IL1B
CDC25A
SERPINB2
GADD45A
CDK6
RBL1
HIRA
H2AFJ
ERBB2
TAF15
FUS-DDIT3
GMNN
CDK4
MCM6
E2F4
DDIT3
HES1
ATF3
MDM2
CCNE2
MYC
SUZ12
EIF1
SNAIL1
H2AFX
FUS (C-terminal)
HIST2H2AA3
CDKN1A
CCNE1
CCND1
POLR2B
ATF6
E2F1
RUNX1
CEBPG
E2F7
RB1
VIM
YWHAZ
PA2G4
CCNA2
MAGOH
IL6
PCNA
CDT1
MMP1
EGR1
RPL7
TSPYL4
ATF4
HIST1H2AC
EWSR1
CDK2
ACTB
FUS (N-terminal)
IL8
RPS10
RPS26
HIST1H2AE
GAPDH
CCNB2
POU5F1
ND1
CBX3
CCNB1
Gene importance

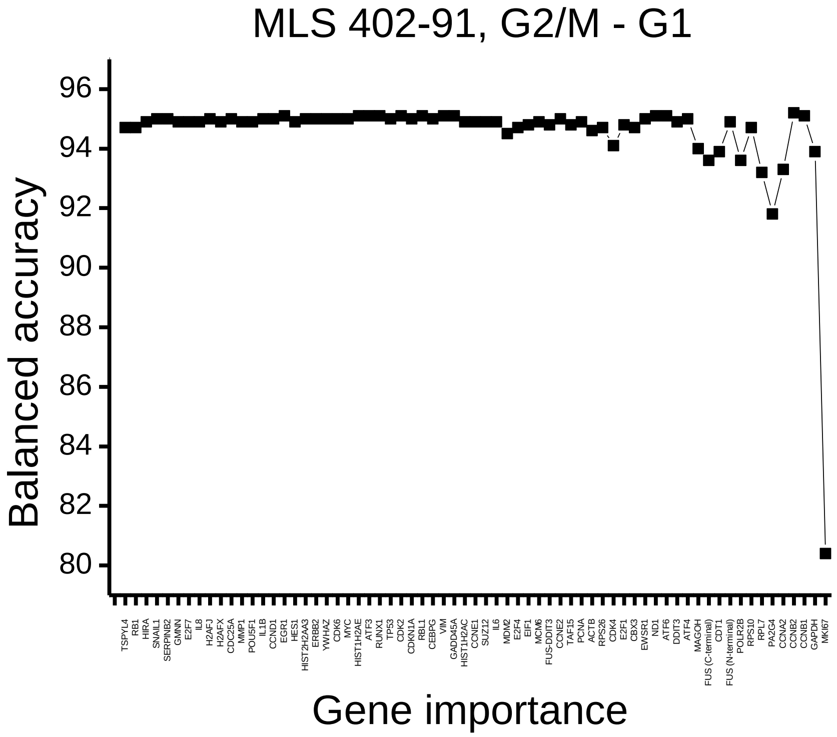

MLS 402-91, G2/M - G1
Balanced accuracy
96
94
92
90
88
86
84
82
80
TSPYL4
RB1
HIRA
SNAIL1
SERPINB2
GMNN
E2F7
IL8
H2AFJ
H2AFX
CDC25A
MMP1
POU5F1
IL1B
CCND1
EGR1
HES1
HIST2H2AA3
ERBB2
YWHAZ
CDK6
MYC
HIST1H2AE
ATF3
RUNX1
TP53
CDK2
CDKN1A
RBL1
CEBPG
VIM
GADD45A
HIST1H2AC
CCNE1
SUZ12
IL6
MDM2
E2F4
EIF1
MCM6
FUS-DDIT3
CCNE2
TAF15
PCNA
ACTB
RPS26
CDK4
E2F1
CBX3
EWSR1
ND1
ATF6
DDIT3
ATF4
MAGOH
FUS (C-terminal)
CDT1
FUS (N-terminal)
POLR2B
RPS10
RPL7
PA2G4
CCNA2
CCNB2
CCNB1
GAPDH
MKI67
Gene importance

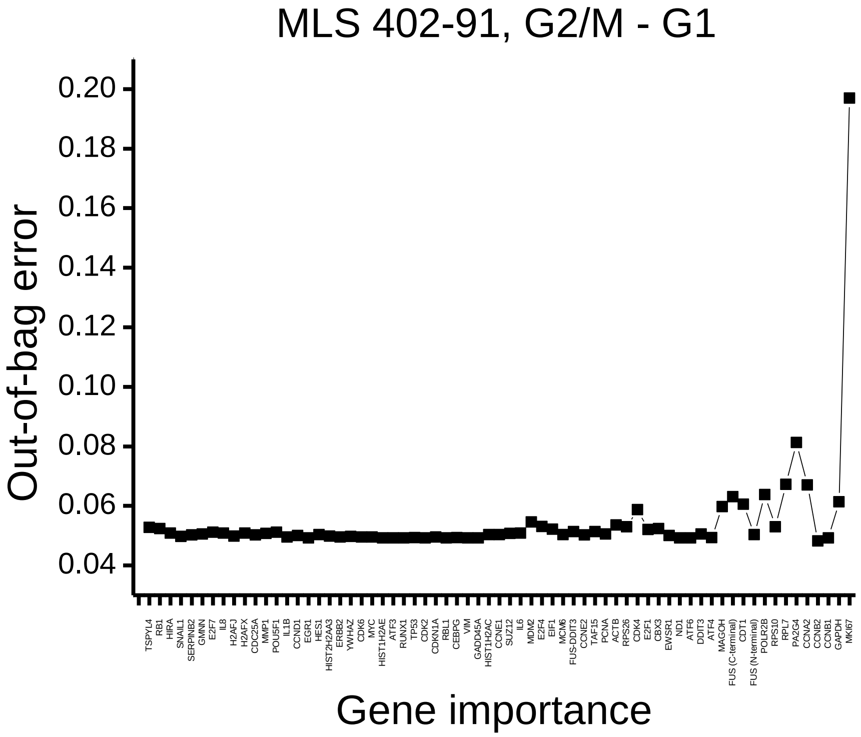
MLS 402-91, G2/M - G1
Out-of-bag error
0.20
0.18
0.16
0.14
0.12
0.10
0.08
0.06
0.04
TSPYL4
RB1
HIRA
SNAIL1
SERPINB2
GMNN
E2F7
IL8
H2AFJ
H2AFX
CDC25A
MMP1
POU5F1
IL1B
CCND1
EGR1
HES1
HIST2H2AA3
ERBB2
YWHAZ
CDK6
MYC
HIST1H2AE
ATF3
RUNX1
TP53
CDK2
CDKN1A
RBL1
CEBPG
VIM
GADD45A
HIST1H2AC
CCNE1
SUZ12
IL6
MDM2
E2F4
EIF1
MCM6
FUS-DDIT3
CCNE2
TAF15
PCNA
ACTB
RPS26
CDK4
E2F1
CBX3
EWSR1
ND1
ATF6
DDIT3
ATF4
MAGOH
FUS (C-terminal)
CDT1
FUS (N-terminal)
POLR2B
RPS10
RPL7
PA2G4
CCNA2
CCNB2
CCNB1
GAPDH
MKI67
Gene importance

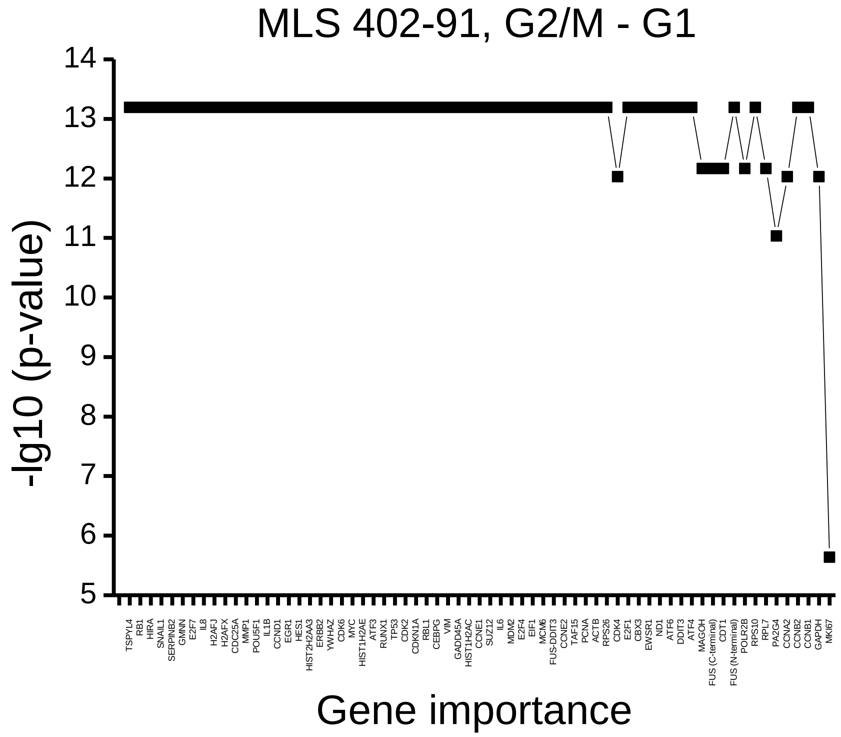
MLS 402-91, G2/M - G1
-lg10 (p-value)
14
13
12
11
10
9
8
7
6
5
TSPYL4
RB1
HIRA
SNAIL1
SERPINB2
GMNN
E2F7
IL8
H2AFJ
H2AFX
CDC25A
MMP1
POU5F1
IL1B
CCND1
EGR1
HES1
HIST2H2AA3
ERBB2
YWHAZ
CDK6
MYC
HIST1H2AE
ATF3
RUNX1
TP53
CDK2
CDKN1A
RBL1
CEBPG
VIM
GADD45A
HIST1H2AC
CCNE1
SUZ12
IL6
MDM2
E2F4
EIF1
MCM6
FUS-DDIT3
CCNE2
TAF15
PCNA
ACTB
RPS26
CDK4
E2F1
CBX3
EWSR1
ND1
ATF6
DDIT3
ATF4
MAGOH
FUS (C-terminal)
CDT1
FUS (N-terminal)
POLR2B
RPS10
RPL7
PA2G4
CCNA2
CCNB2
CCNB1
GAPDH
MKI67
Gene importance

MCF7

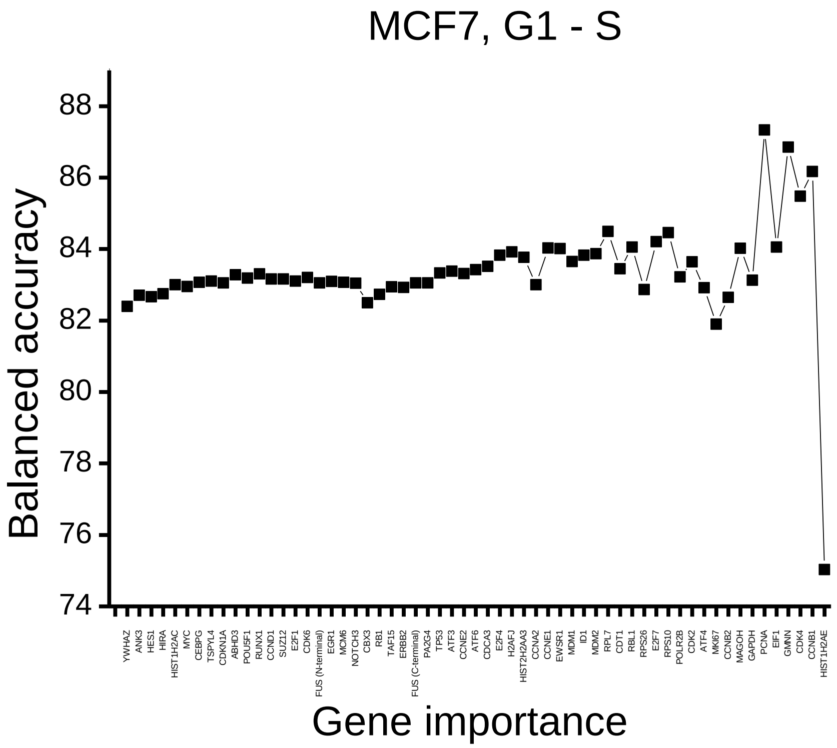
MCF7, G1 - S
Balanced accuracy
88
86
84
82
80
78
76
74
YWHAZ
ANK3
HES1
HIRA
HIST1H2AC
MYC
CEBPG
TSPYL4
CDKN1A
ABHD3
POU5F1
RUNX1
CCND1
SUZ12
E2F1
CDK6
FUS (N-terminal)
EGR1
MCM6
NOTCH3
CBX3
RB1
TAF15
ERBB2
FUS (C-terminal)
PA2G4
TP53
ATF3
CCNE2
ATF6
CDCA3
E2F4
H2AFJ
HIST2H2AA3
CCNA2
CCNE1
EWSR1
MDM1
ID1
MDM2
RPL7
CDT1
RBL1
RPS26
E2F7
RPS10
POLR2B
CDK2
ATF4
MKI67
CCNB2
MAGOH
GAPDH
PCNA
EIF1
GMNN
CDK4
CCNB1
HIST1H2AE
Gene importance

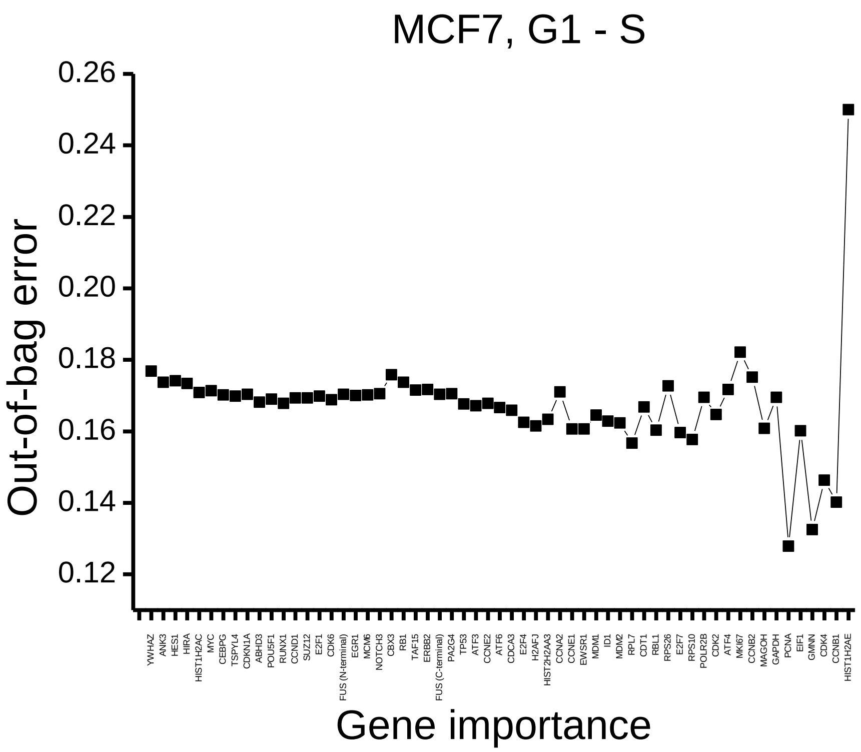
MCF7, G1 - S
Out-of-bag error
0.26
0.24
0.22
0.20
0.18
0.16
0.14
0.12
YWHAZ
ANK3
HES1
HIRA
HIST1H2AC
MYC
CEBPG
TSPYL4
CDKN1A
ABHD3
POU5F1
RUNX1
CCND1
SUZ12
E2F1
CDK6
FUS (N-terminal)
EGR1
MCM6
NOTCH3
CBX3
RB1
TAF15
ERBB2
FUS (C-terminal)
PA2G4
TP53
ATF3
CCNE2
ATF6
CDCA3
E2F4
H2AFJ
HIST2H2AA3
CCNA2
CCNE1
EWSR1
MDM1
ID1
MDM2
RPL7
CDT1
RBL1
RPS26
E2F7
RPS10
POLR2B
CDK2
ATF4
MKI67
CCNB2
MAGOH
GAPDH
PCNA
EIF1
GMNN
CDK4
CCNB1
HIST1H2AE
Gene importance

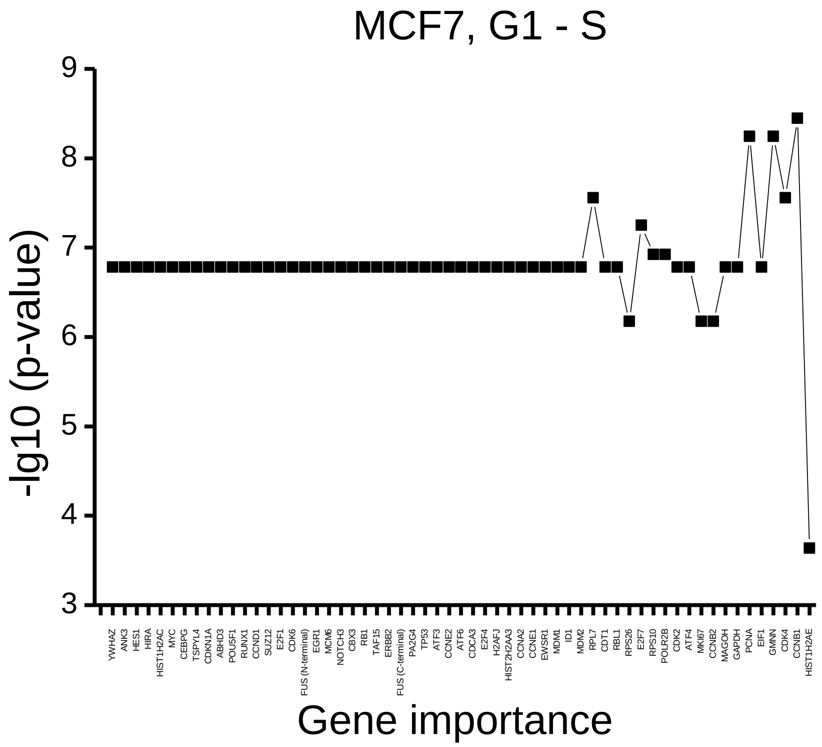
MCF7, G1 - S
-lg10 (p-value)
9
8
7
6
5
4
3
YWHAZ
ANK3
HES1
HIRA
HIST1H2AC
MYC
CEBPG
TSPYL4
CDKN1A
ABHD3
POU5F1
RUNX1
CCND1
SUZ12
E2F1
CDK6
FUS (N-terminal)
EGR1
MCM6
NOTCH3
CBX3
RB1
TAF15
ERBB2
FUS (C-terminal)
PA2G4
TP53
ATF3
CCNE2
ATF6
CDCA3
E2F4
H2AFJ
HIST2H2AA3
CCNA2
CCNE1
EWSR1
MDM1
ID1
MDM2
RPL7
CDT1
RBL1
RPS26
E2F7
RPS10
POLR2B
CDK2
ATF4
MKI67
CCNB2
MAGOH
GAPDH
PCNA
EIF1
GMNN
CDK4
CCNB1
HIST1H2AE
Gene importance

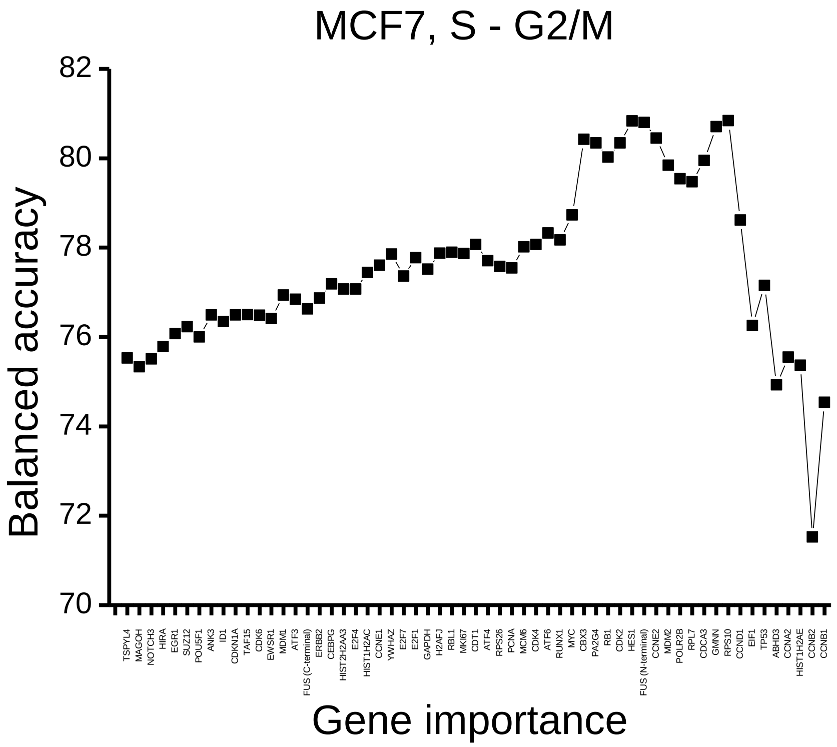
MCF7, S - G2/M
Balanced accuracy
82
80
78
76
74
72
70
TSPYL4
MAGOH
NOTCH3
HIRA
EGR1
SUZ12
POU5F1
ANK3
ID1
CDKN1A
TAF15
CDK6
EWSR1
MDM1
ATF3
FUS (C-terminal)
ERBB2
CEBPG
HIST2H2AA3
E2F4
HIST1H2AC
CCNE1
YWHAZ
E2F7
E2F1
GAPDH
H2AFJ
RBL1
MKI67
CDT1
ATF4
RPS26
PCNA
MCM6
CDK4
ATF6
RUNX1
MYC
CBX3
PA2G4
RB1
CDK2
HES1
FUS (N-terminal)
CCNE2
MDM2
POLR2B
RPL7
CDCA3
GMNN
RPS10
CCND1
EIF1
TP53
ABHD3
CCNA2
HIST1H2AE
CCNB2
CCNB1
Gene importance

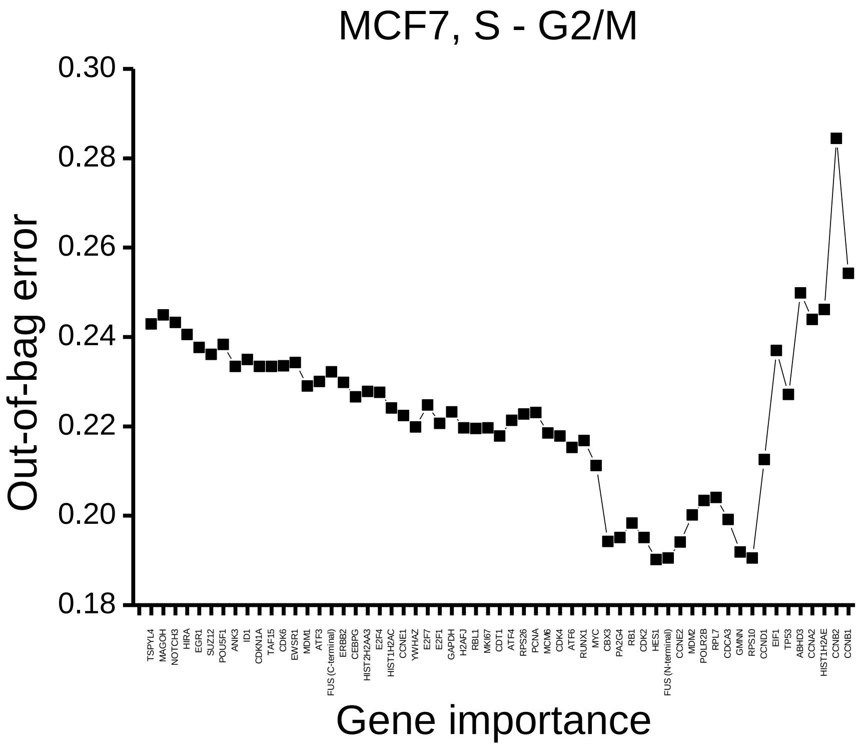
MCF7, S - G2/M
Out-of-bag error
0.30
0.28
0.26
0.24
0.22
0.20
0.18
TSPYL4
MAGOH
NOTCH3
HIRA
EGR1
SUZ12
POU5F1
ANK3
ID1
CDKN1A
TAF15
CDK6
EWSR1
MDM1
ATF3
FUS (C-terminal)
ERBB2
CEBPG
HIST2H2AA3
E2F4
HIST1H2AC
CCNE1
YWHAZ
E2F7
E2F1
GAPDH
H2AFJ
RBL1
MKI67
CDT1
ATF4
RPS26
PCNA
MCM6
CDK4
ATF6
RUNX1
MYC
CBX3
PA2G4
RB1
CDK2
HES1
FUS (N-terminal)
CCNE2
MDM2
POLR2B
RPL7
CDCA3
GMNN
RPS10
CCND1
EIF1
TP53
ABHD3
CCNA2
HIST1H2AE
CCNB2
CCNB1
Gene importance

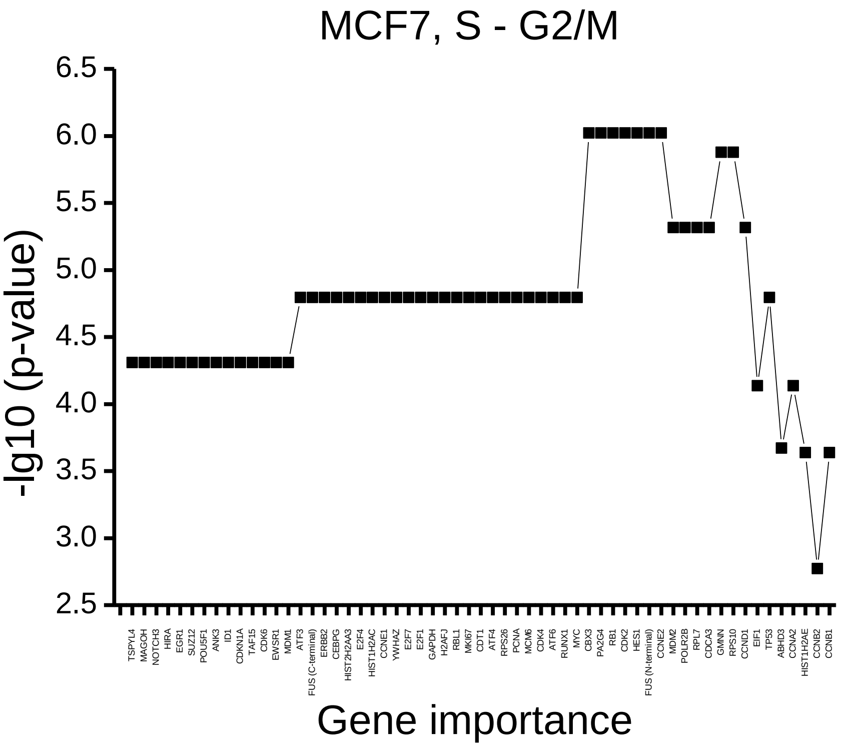
MCF7, S - G2/M
-lg10 (p-value)
6.5
6.0
5.5
5.0
4.5
4.0
3.5
3.0
2.5
TSPYL4
MAGOH
NOTCH3
HIRA
EGR1
SUZ12
POU5F1
ANK3
ID1
CDKN1A
TAF15
CDK6
EWSR1
MDM1
ATF3
FUS (C-terminal)
ERBB2
CEBPG
HIST2H2AA3
E2F4
HIST1H2AC
CCNE1
YWHAZ
E2F7
E2F1
GAPDH
H2AFJ
RBL1
MKI67
CDT1
ATF4
RPS26
PCNA
MCM6
CDK4
ATF6
RUNX1
MYC
CBX3
PA2G4
RB1
CDK2
HES1
FUS (N-terminal)
CCNE2
MDM2
POLR2B
RPL7
CDCA3
GMNN
RPS10
CCND1
EIF1
TP53
ABHD3
CCNA2
HIST1H2AE
CCNB2
CCNB1
Gene importance

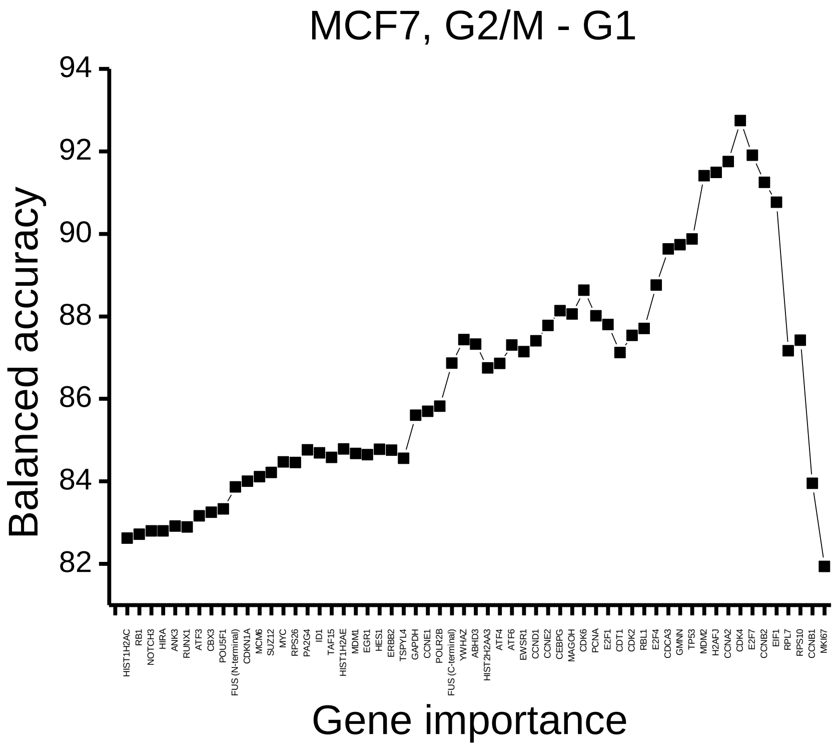
MCF7, G2/M - G1
Balanced accuracy
94
92
90
88
86
84
82
HIST1H2AC
RB1
NOTCH3
HIRA
ANK3
RUNX1
ATF3
CBX3
POU5F1
FUS (N-terminal)
CDKN1A
MCM6
SUZ12
MYC
RPS26
PA2G4
ID1
TAF15
HIST1H2AE
MDM1
EGR1
HES1
ERBB2
TSPYL4
GAPDH
CCNE1
POLR2B
FUS (C-terminal)
YWHAZ
ABHD3
HIST2H2AA3
ATF4
ATF6
EWSR1
CCND1
CCNE2
CEBPG
MAGOH
CDK6
PCNA
E2F1
CDT1
CDK2
RBL1
E2F4
CDCA3
GMNN
TP53
MDM2
H2AFJ
CCNA2
CDK4
E2F7
CCNB2
EIF1
RPL7
RPS10
CCNB1
MKI67
Gene importance

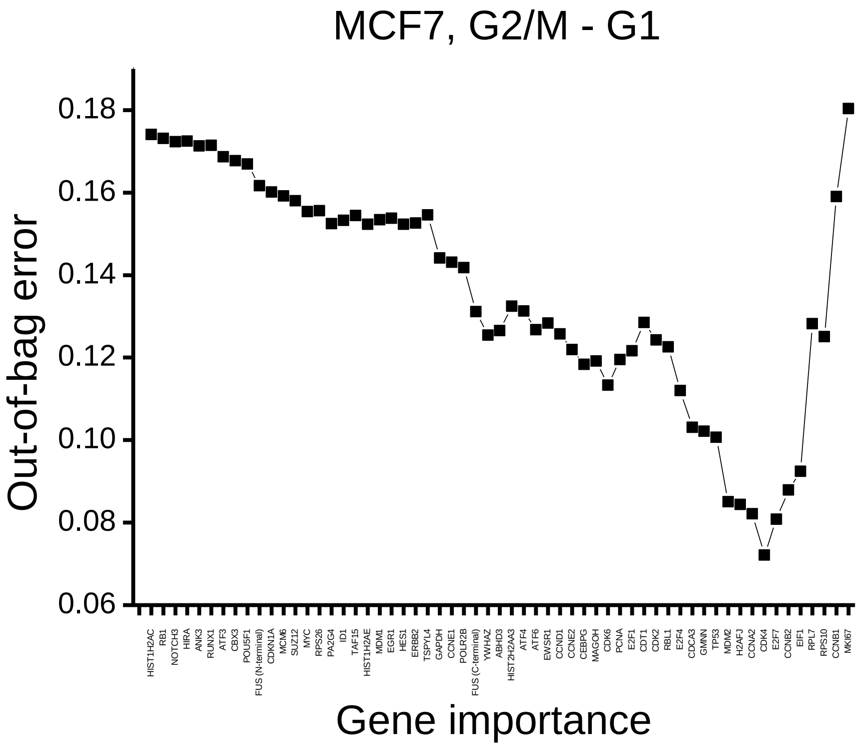
MCF7, G2/M - G1
Out-of-bag error
0.18
0.16
0.14
0.12
0.10
0.08
0.06
HIST1H2AC
RB1
NOTCH3
HIRA
ANK3
RUNX1
ATF3
CBX3
POU5F1
FUS (N-terminal)
CDKN1A
MCM6
SUZ12
MYC
RPS26
PA2G4
ID1
TAF15
HIST1H2AE
MDM1
EGR1
HES1
ERBB2
TSPYL4
GAPDH
CCNE1
POLR2B
FUS (C-terminal)
YWHAZ
ABHD3
HIST2H2AA3
ATF4
ATF6
EWSR1
CCND1
CCNE2
CEBPG
MAGOH
CDK6
PCNA
E2F1
CDT1
CDK2
RBL1
E2F4
CDCA3
GMNN
TP53
MDM2
H2AFJ
CCNA2
CDK4
E2F7
CCNB2
EIF1
RPL7
RPS10
CCNB1
MKI67
Gene importance

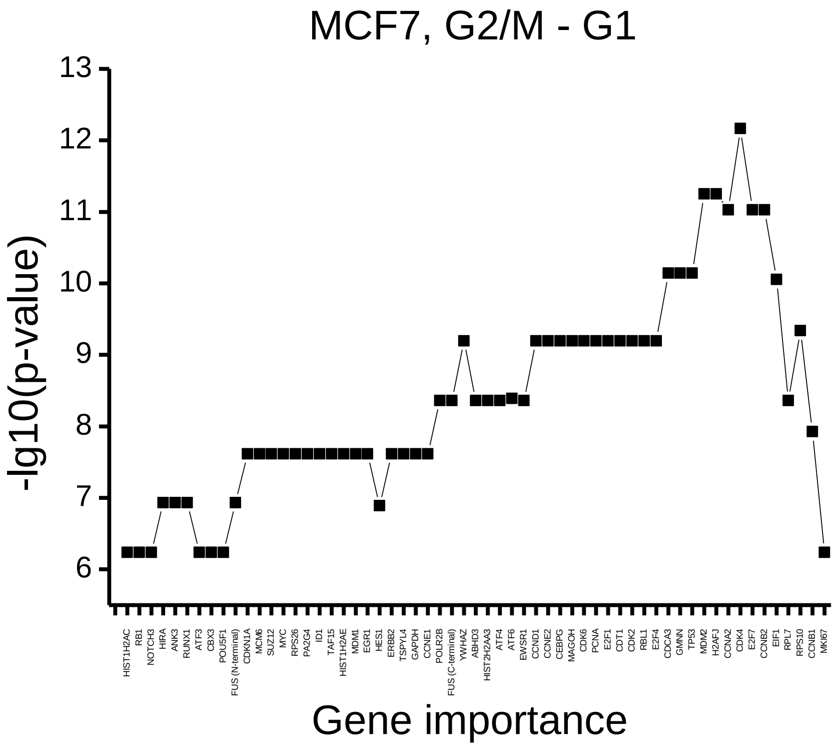
MCF7, G2/M - G1
-lg10(p-value)
13
12
11
10
9
8
7
6
HIST1H2AC
RB1
NOTCH3
HIRA
ANK3
RUNX1
ATF3
CBX3
POU5F1
FUS (N-terminal)
CDKN1A
MCM6
SUZ12
MYC
RPS26
PA2G4
ID1
TAF15
HIST1H2AE
MDM1
EGR1
HES1
ERBB2
TSPYL4
GAPDH
CCNE1
POLR2B
FUS (C-terminal)
YWHAZ
ABHD3
HIST2H2AA3
ATF4
ATF6
EWSR1
CCND1
CCNE2
CEBPG
MAGOH
CDK6
PCNA
E2F1
CDT1
CDK2
RBL1
E2F4
CDCA3
GMNN
TP53
MDM2
H2AFJ
CCNA2
CDK4
E2F7
CCNB2
EIF1
RPL7
RPS10
CCNB1
MKI67
Gene importance

MSC

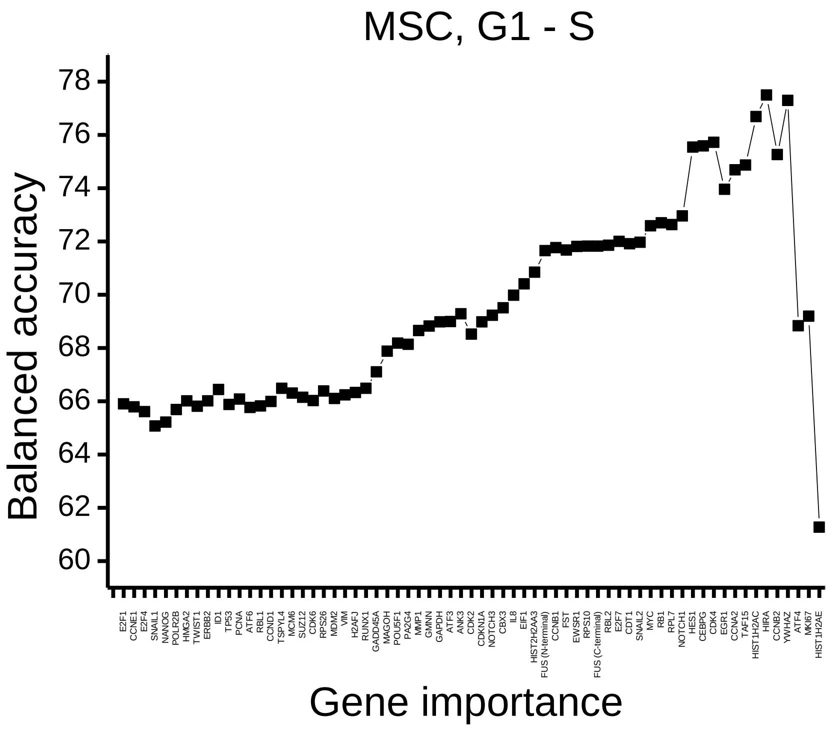
MSC, G1 - S
Balanced accuracy
78
76
74
72
70
68
66
64
62
60
E2F1
CCNE1
E2F4
SNAIL1
NANOG
POLR2B
HMGA2
TWIST1
ERBB2
ID1
TP53
PCNA
ATF6
RBL1
CCND1
TSPYL4
MCM6
SUZ12
CDK6
RPS26
MDM2
VIM
H2AFJ
RUNX1
GADD45A
MAGOH
POU5F1
PA2G4
MMP1
GMNN
GAPDH
ATF3
ANK3
CDK2
CDKN1A
NOTCH3
CBX3
IL8
EIF1
HIST2H2AA3
FUS (N-terminal)
CCNB1
FST
EWSR1
RPS10
FUS (C-terminal)
RBL2
E2F7
CDT1
SNAIL2
MYC
RB1
RPL7
NOTCH1
HES1
CEBPG
CDK4
EGR1
CCNA2
TAF15
HIST1H2AC
HIRA
CCNB2
YWHAZ
ATF4
MKI67
HIST1H2AE
Gene importance

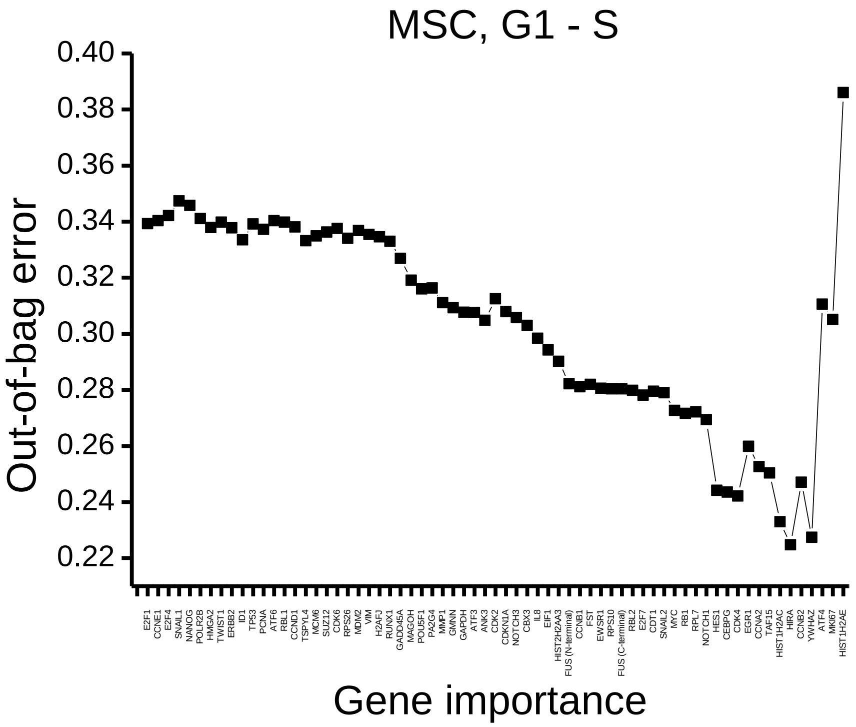
MSC, G1 - S
Out-of-bag error
0.40
0.38
0.36
0.34
0.32
0.30
0.28
0.26
0.24
0.22
E2F1
CCNE1
E2F4
SNAIL1
NANOG
POLR2B
HMGA2
TWIST1
ERBB2
ID1
TP53
PCNA
ATF6
RBL1
CCND1
TSPYL4
MCM6
SUZ12
CDK6
RPS26
MDM2
VIM
H2AFJ
RUNX1
GADD45A
MAGOH
POU5F1
PA2G4
MMP1
GMNN
GAPDH
ATF3
ANK3
CDK2
CDKN1A
NOTCH3
CBX3
IL8
EIF1
HIST2H2AA3
FUS (N-terminal)
CCNB1
FST
EWSR1
RPS10
FUS (C-terminal)
RBL2
E2F7
CDT1
SNAIL2
MYC
RB1
RPL7
NOTCH1
HES1
CEBPG
CDK4
EGR1
CCNA2
TAF15
HIST1H2AC
HIRA
CCNB2
YWHAZ
ATF4
MKI67
HIST1H2AE
Gene importance

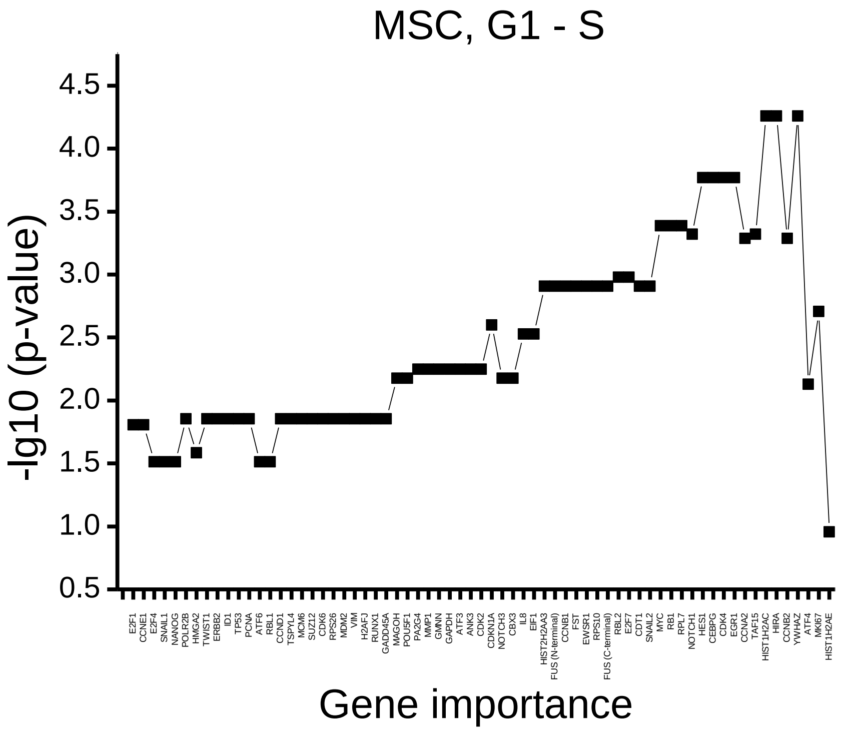
MSC, G1 - S
-lg10 (p-value)
4.5
4.0
3.5
3.0
2.5
2.0
1.5
1.0
0.5
E2F1
CCNE1
E2F4
SNAIL1
NANOG
POLR2B
HMGA2
TWIST1
ERBB2
ID1
TP53
PCNA
ATF6
RBL1
CCND1
TSPYL4
MCM6
SUZ12
CDK6
RPS26
MDM2
VIM
H2AFJ
RUNX1
GADD45A
MAGOH
POU5F1
PA2G4
MMP1
GMNN
GAPDH
ATF3
ANK3
CDK2
CDKN1A
NOTCH3
CBX3
IL8
EIF1
HIST2H2AA3
FUS (N-terminal)
CCNB1
FST
EWSR1
RPS10
FUS (C-terminal)
RBL2
E2F7
CDT1
SNAIL2
MYC
RB1
RPL7
NOTCH1
HES1
CEBPG
CDK4
EGR1
CCNA2
TAF15
HIST1H2AC
HIRA
CCNB2
YWHAZ
ATF4
MKI67
HIST1H2AE
Gene importance

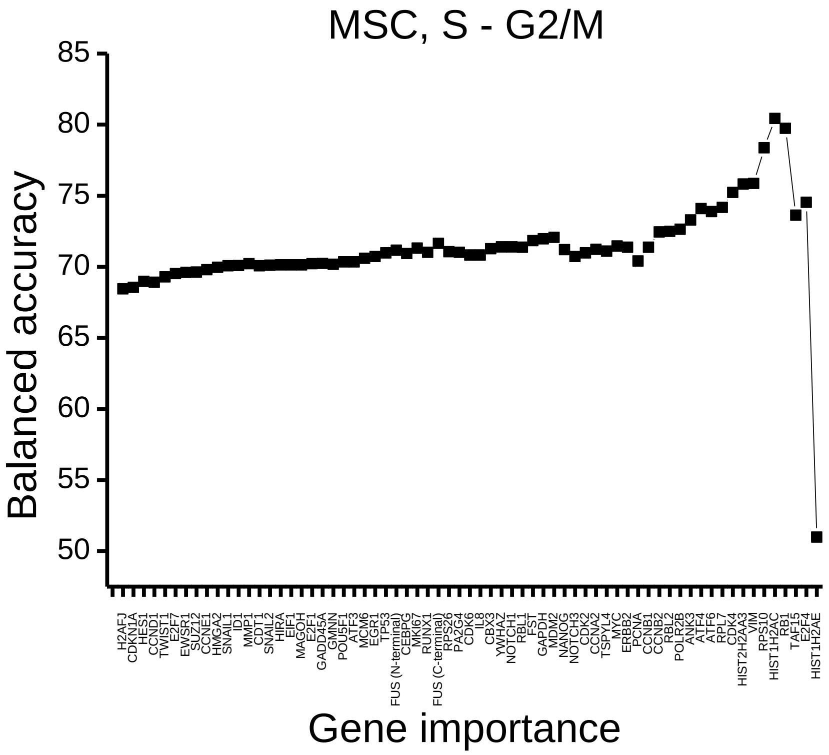
MSC, S - G2/M
Balanced accuracy
85
80
75
70
65
60
55
50
H2AFJ
CDKN1A
HES1
CCND1
TWIST1
E2F7
EWSR1
SUZ12
CCNE1
HMGA2
SNAIL1
ID1
MMP1
CDT1
SNAIL2
HIRA
EIF1
MAGOH
E2F1
GADD45A
GMNN
POU5F1
ATF3
MCM6
EGR1
TP53
FUS (N-terminal)
CEBPG
MKI67
RUNX1
FUS (C-terminal)
RPS26
PA2G4
CDK6
IL8
CBX3
YWHAZ
NOTCH1
RBL1
FST
GAPDH
MDM2
NANOG
NOTCH3
CDK2
CCNA2
TSPYL4
MYC
ERBB2
PCNA
CCNB1
CCNB2
RBL2
POLR2B
ANK3
ATF4
ATF6
RPL7
CDK4
HIST2H2AA3
VIM
RPS10
HIST1H2AC
RB1
TAF15
E2F4
HIST1H2AE
Gene importance

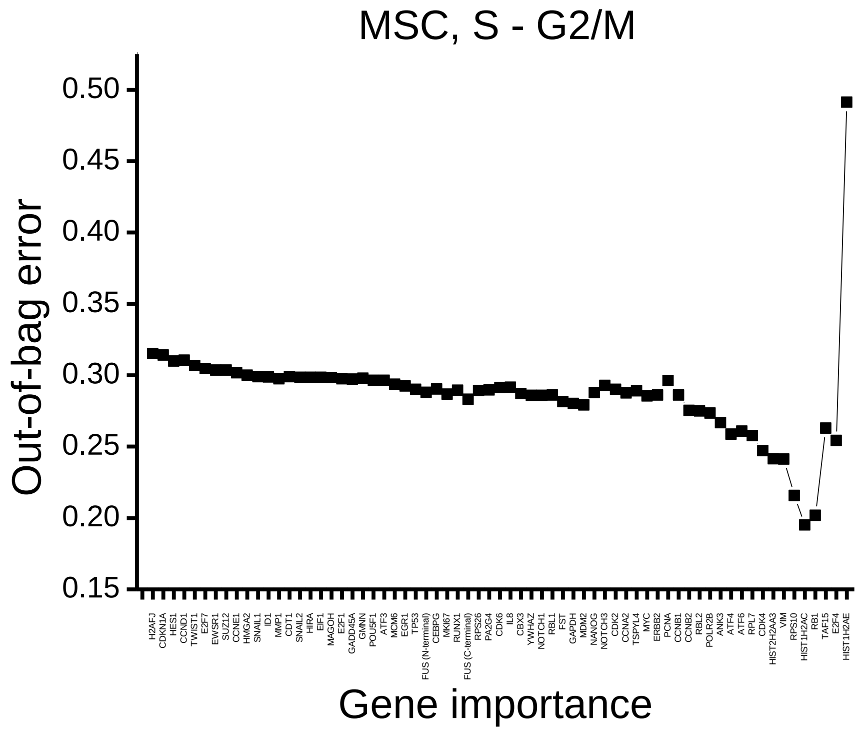
MSC, S - G2/M
Out-of-bag error
0.50
0.45
0.40
0.35
0.30
0.25
0.20
0.15
H2AFJ
CDKN1A
HES1
CCND1
TWIST1
E2F7
EWSR1
SUZ12
CCNE1
HMGA2
SNAIL1
ID1
MMP1
CDT1
SNAIL2
HIRA
EIF1
MAGOH
E2F1
GADD45A
GMNN
POU5F1
ATF3
MCM6
EGR1
TP53
FUS (N-terminal)
CEBPG
MKI67
RUNX1
FUS (C-terminal)
RPS26
PA2G4
CDK6
IL8
CBX3
YWHAZ
NOTCH1
RBL1
FST
GAPDH
MDM2
NANOG
NOTCH3
CDK2
CCNA2
TSPYL4
MYC
ERBB2
PCNA
CCNB1
CCNB2
RBL2
POLR2B
ANK3
ATF4
ATF6
RPL7
CDK4
HIST2H2AA3
VIM
RPS10
HIST1H2AC
RB1
TAF15
E2F4
HIST1H2AE
Gene importance

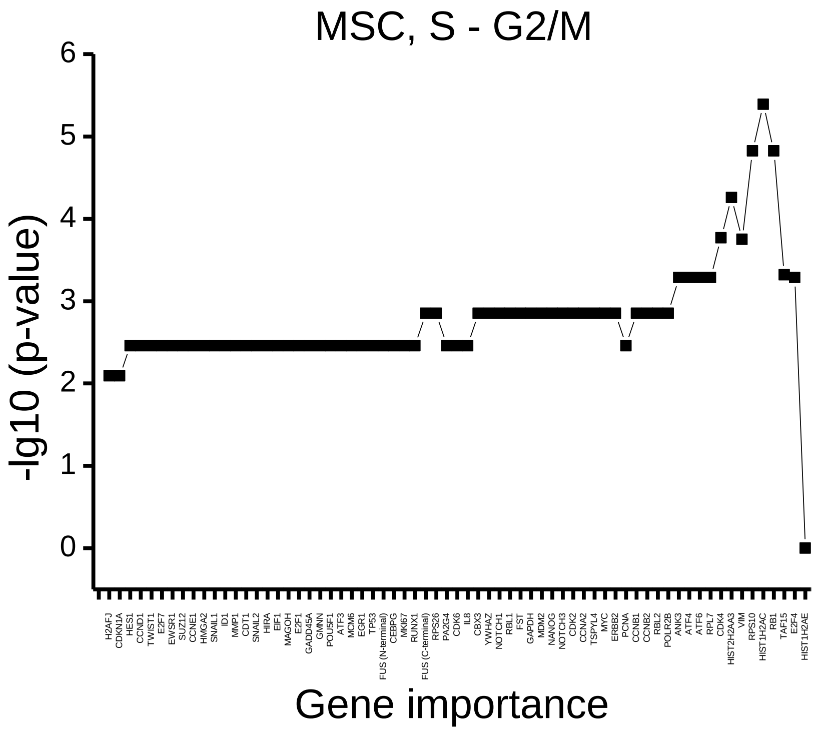

MSC, S - G2/M
-lg10 (p-value)
6
5
4
3
2
1
0
H2AFJ
CDKN1A
HES1
CCND1
TWIST1
E2F7
EWSR1
SUZ12
CCNE1
HMGA2
SNAIL1
ID1
MMP1
CDT1
SNAIL2
HIRA
EIF1
MAGOH
E2F1
GADD45A
GMNN
POU5F1
ATF3
MCM6
EGR1
TP53
FUS (N-terminal)
CEBPG
MKI67
RUNX1
FUS (C-terminal)
RPS26
PA2G4
CDK6
IL8
CBX3
YWHAZ
NOTCH1
RBL1
FST
GAPDH
MDM2
NANOG
NOTCH3
CDK2
CCNA2
TSPYL4
MYC
ERBB2
PCNA
CCNB1
CCNB2
RBL2
POLR2B
ANK3
ATF4
ATF6
RPL7
CDK4
HIST2H2AA3
VIM
RPS10
HIST1H2AC
RB1
TAF15
E2F4
HIST1H2AE
Gene importance

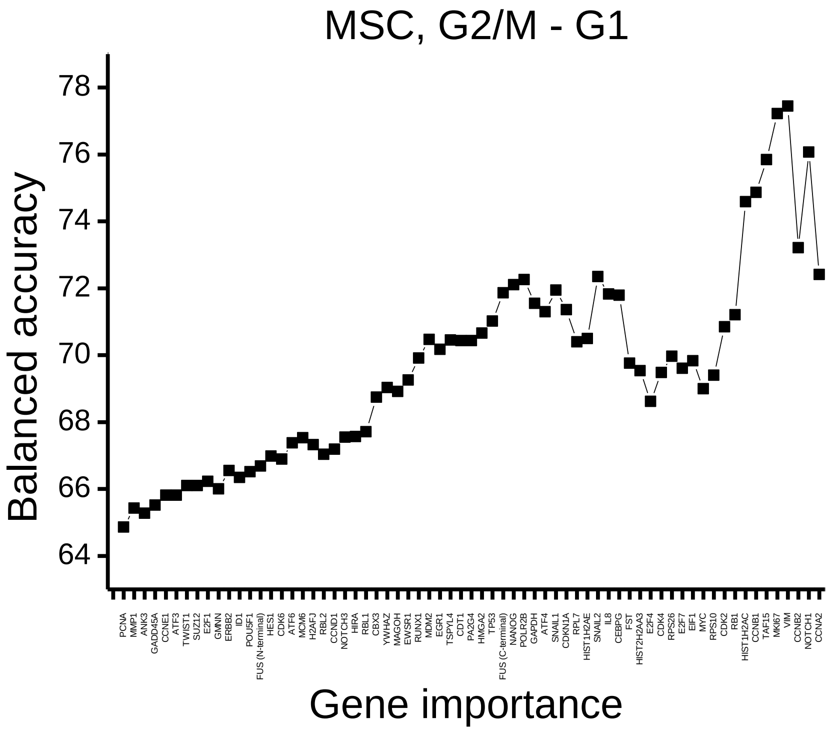
MSC, G2/M - G1
Balanced accuracy
78
76
74
72
70
68
66
64
PCNA
MMP1
ANK3
GADD45A
CCNE1
ATF3
TWIST1
SUZ12
E2F1
GMNN
ERBB2
ID1
POU5F1
FUS (N-terminal)
HES1
CDK6
ATF6
MCM6
H2AFJ
RBL2
CCND1
NOTCH3
HIRA
RBL1
CBX3
YWHAZ
MAGOH
EWSR1
RUNX1
MDM2
EGR1
TSPYL4
CDT1
PA2G4
HMGA2
TP53
FUS (C-terminal)
NANOG
POLR2B
GAPDH
ATF4
SNAIL1
CDKN1A
RPL7
HIST1H2AE
SNAIL2
IL8
CEBPG
FST
HIST2H2AA3
E2F4
CDK4
RPS26
E2F7
EIF1
MYC
RPS10
CDK2
RB1
HIST1H2AC
CCNB1
TAF15
MKI67
VIM
CCNB2
NOTCH1
CCNA2
Gene importance

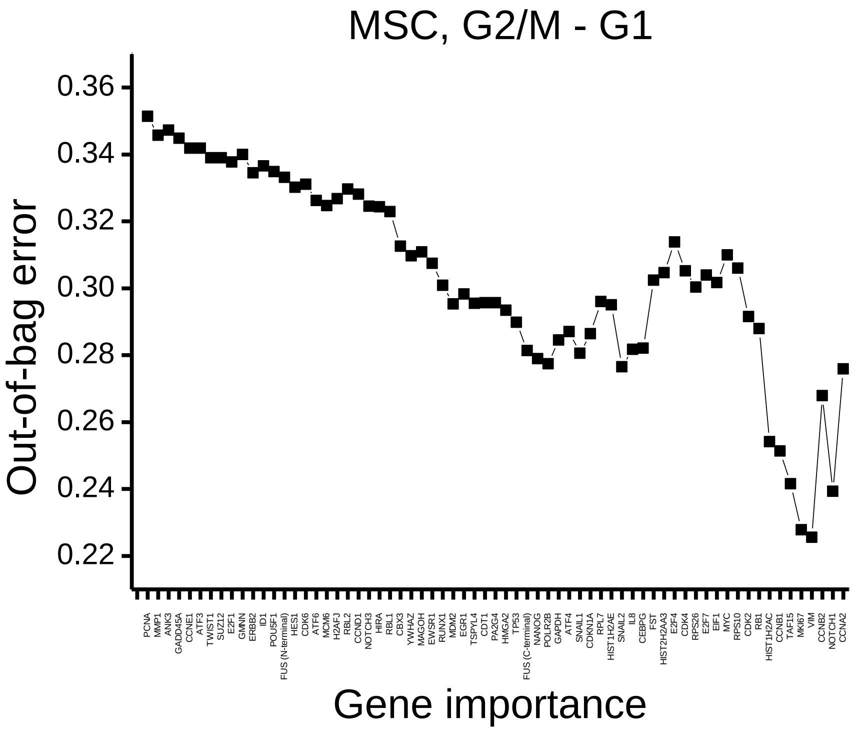
MSC, G2/M - G1
Out-of-bag error
0.36
0.34
0.32
0.30
0.28
0.26
0.24
0.22
PCNA
MMP1
ANK3
GADD45A
CCNE1
ATF3
TWIST1
SUZ12
E2F1
GMNN
ERBB2
ID1
POU5F1
FUS (N-terminal)
HES1
CDK6
ATF6
MCM6
H2AFJ
RBL2
CCND1
NOTCH3
HIRA
RBL1
CBX3
YWHAZ
MAGOH
EWSR1
RUNX1
MDM2
EGR1
TSPYL4
CDT1
PA2G4
HMGA2
TP53
FUS (C-terminal)
NANOG
POLR2B
GAPDH
ATF4
SNAIL1
CDKN1A
RPL7
HIST1H2AE
SNAIL2
IL8
CEBPG
FST
HIST2H2AA3
E2F4
CDK4
RPS26
E2F7
EIF1
MYC
RPS10
CDK2
RB1
HIST1H2AC
CCNB1
TAF15
MKI67
VIM
CCNB2
NOTCH1
CCNA2
Gene importance

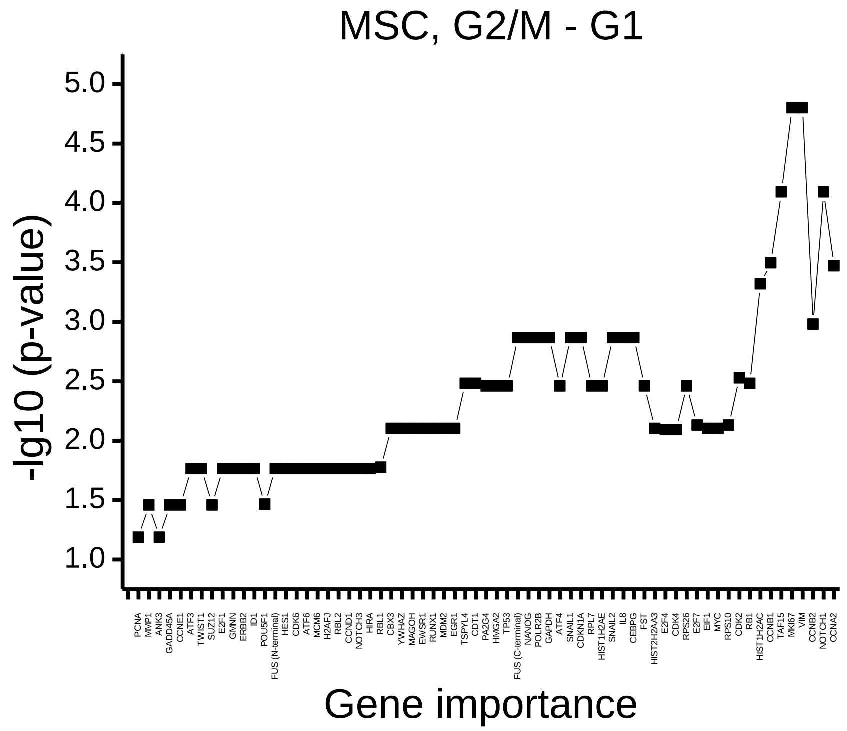

Figure S5. Identification of predictive genes using recursive feature elimination. Balanced accuracy, out-of-bag error and the p-value obtained by applying Fisher's exact test on the confusion matrix are shown. One gene was eliminated at each step until only one gene remained. The sequential list of eliminated genes is indicated on the x-axis. The most important gene is shown to the right in each subfigure.

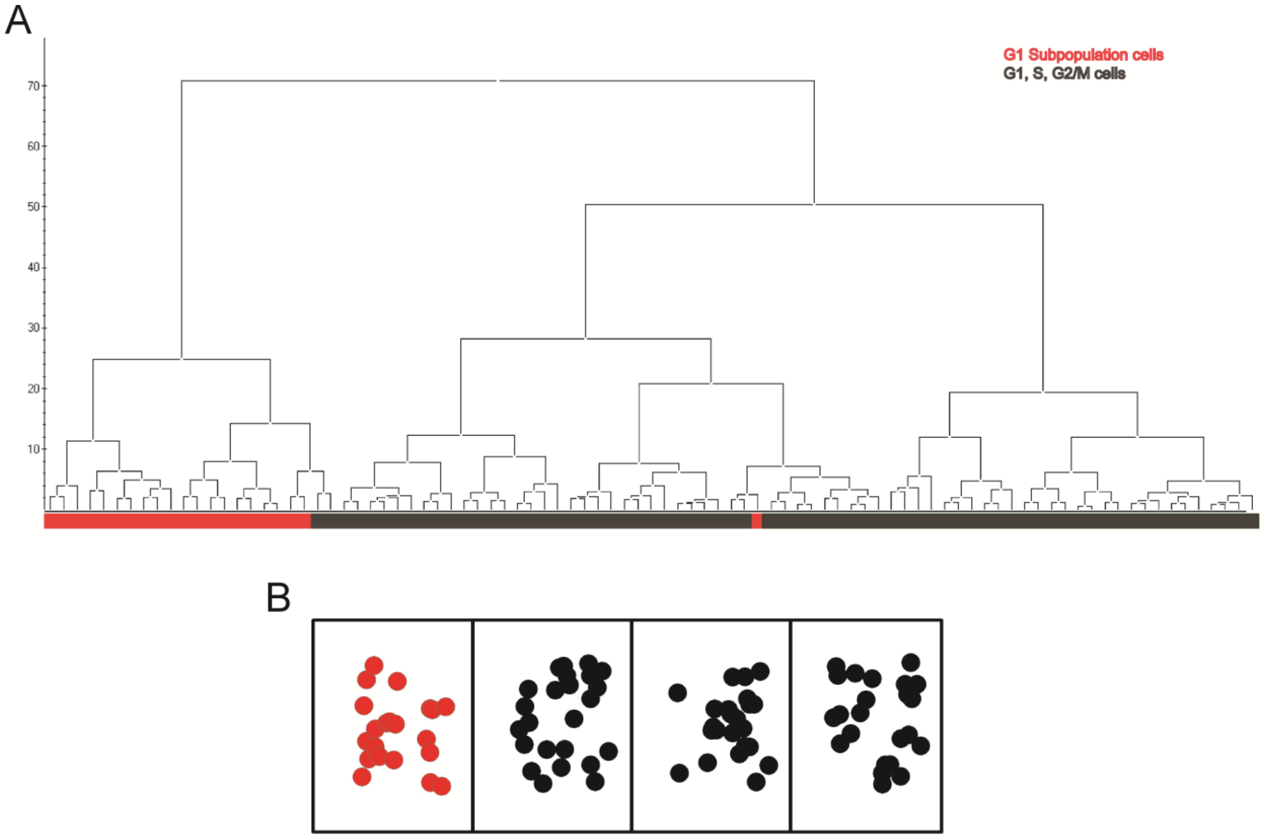

Figure S6. The MLS 402-91 identified G1 subpopulation was validated by other algorithms. The defined subpopulation in MLS 402-91 (Figure 2A) was also identified using **(A)** hierarchical clustering and **(B)** Kohonen self-organizing maps.

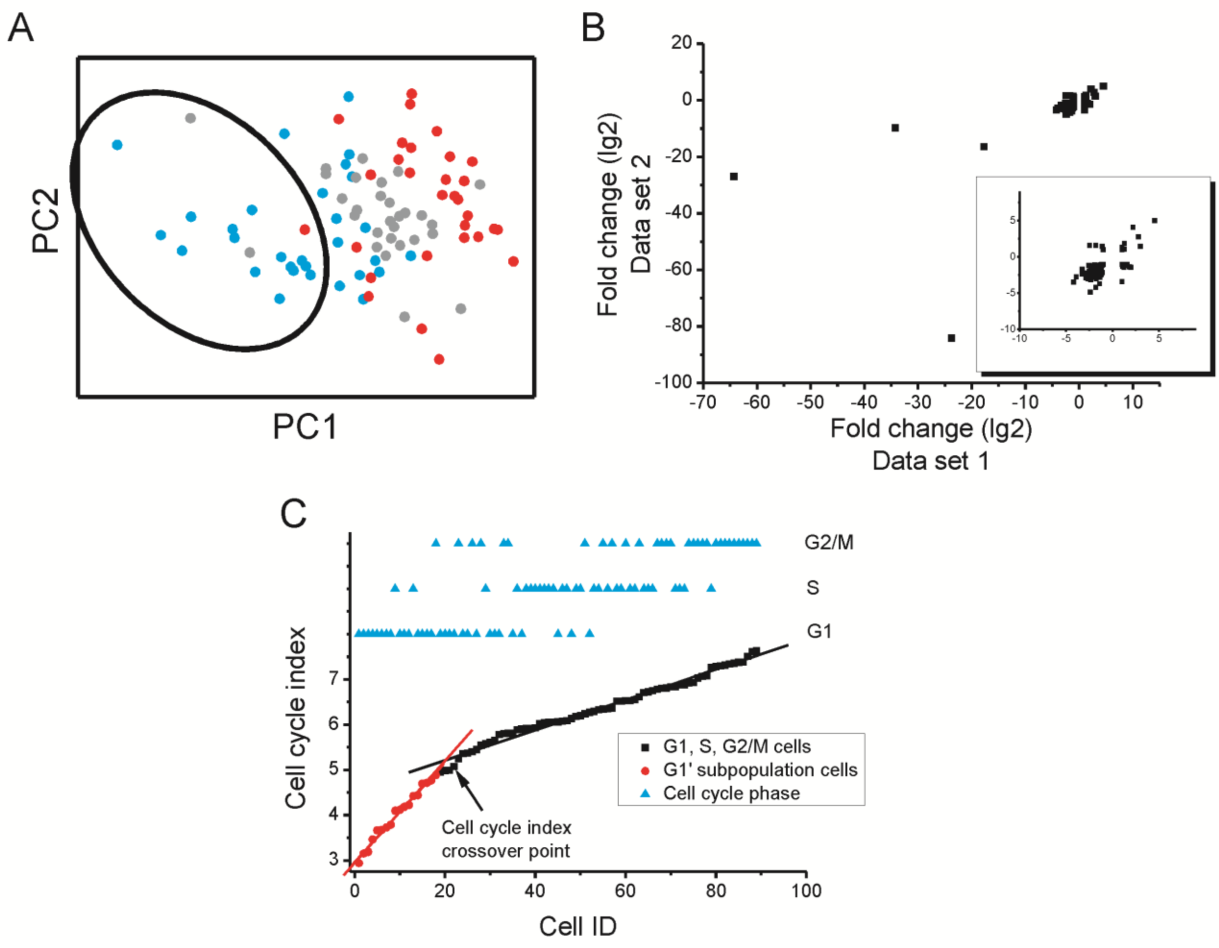

Figure S7. The MLS402-91 identified G1' subpopulation was also detected in an additional data set. The independent MLS 402-91 data set was used to test the reproducibility of the original data set. **(A)** Identification of a G1 specific subpopulation using PCA. **(B)** The gene expression profile of the identified G1' subpopulation was similar for the two MLS 402-91 data sets. **(C)** The cell cycle index of each cell is shown in relation to its cell cycle phase. Subpopulation cells identified in Figure S7A are also indicated. The index was calculated as: (*MKI67* + *RB1* + *HIST1H2AE* + *CCNB1* + *CBX3* + *ND1* + *GAPDH* + *CCNB2* – *E2F1*) / 9, where each gene is represented by its expression in lg2-scale. The cell cycle index crossover point where the index enters a plateau is indicated. The linear fits are shown to guide the eye.

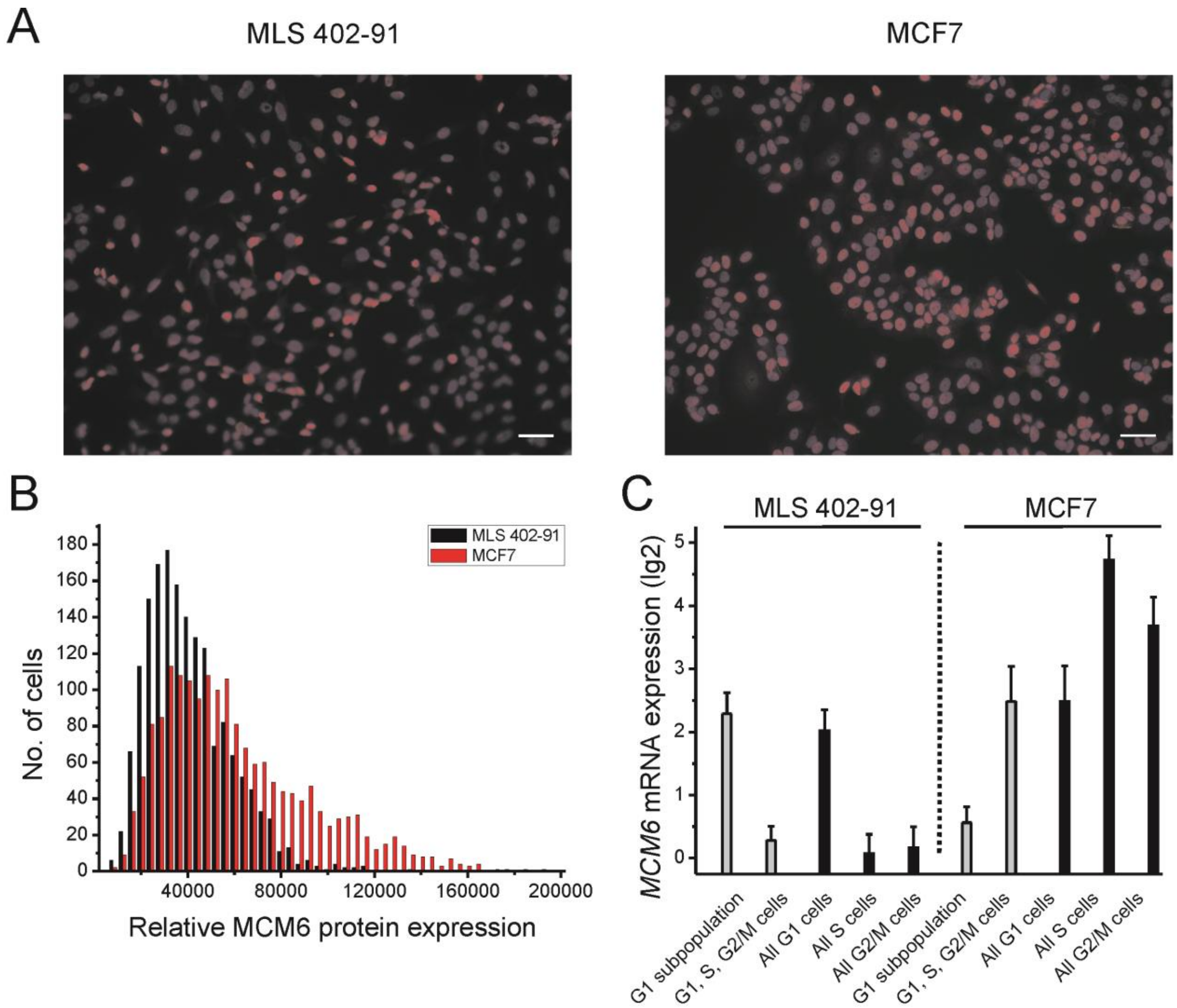

Figure S8. MCM6 protein expression in MLS 402-91 and MCF7. (A) Representative immunofluorescence images of MCM6 expression in MLS 402-91 and MCF7 are shown. Scale bar shows 50 µm. **(B)** Histograms of relative MCM6 protein levels for MLS 402-91 and MCF7. **(C)** *MCM6* mRNA expression. G1 subpopulation and G1, S, G2/M cells (grey) refer to populations defined in Figures 5A and 5D. All G1, S and G2/M cells (black) correspond to both small and large cells in respective cell cycle phase. Mean ±SEM is shown.

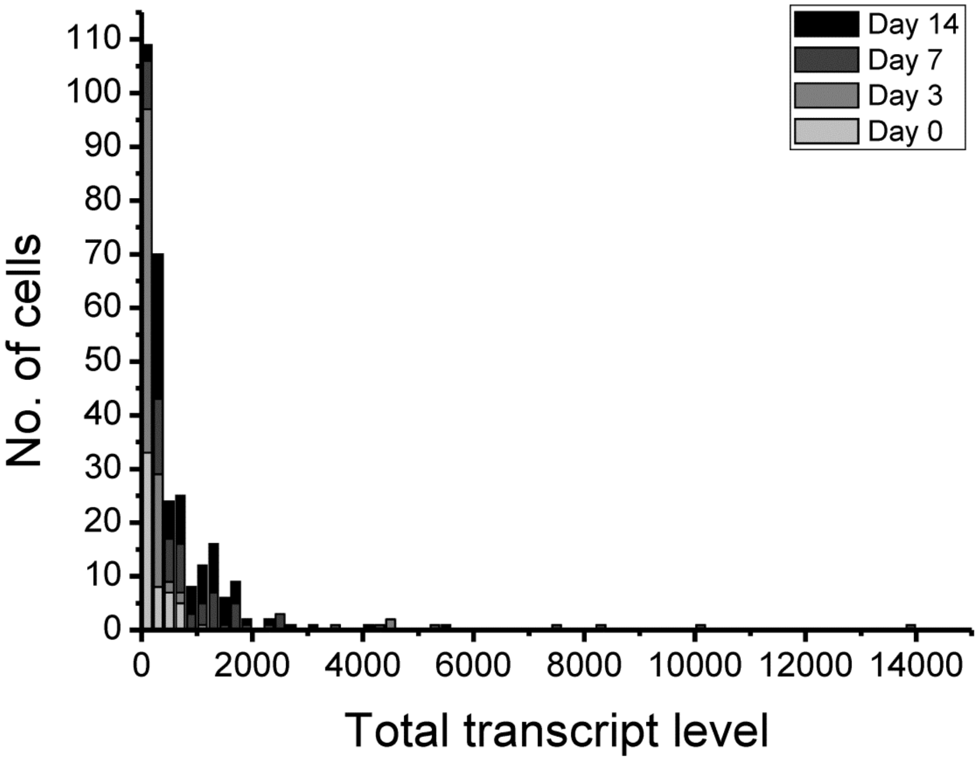

Figure S9. Distribution of total expression levels in individual astrocytes. The total expression levels in 301 individual astrocytes prepared from mice brain before (day 0) and after (day 3, day 7, and day 14) ischemia are shown (Rusnakova et al., 2013). 88% (total number of comparisons = 820) of all pair-wise gene correlation values between individual astrocytes were positive. The data set consisted of 41 genes.
